# Photoenergy harvesting by ammonium molybdate soft hydrogel drops

**DOI:** 10.1038/s41377-025-02016-4

**Published:** 2025-10-21

**Authors:** Zelin Lu, Xinxin Hang, Zinan Zhao, Long Cheng, Yu Zeng, Bixuan Li, Menghan Tian, Baolei Liu, Xuchen Shan, Hongyan Zhu, Zhiying Wang, Menghao Ma, Jinliang Wang, Yongji Gong, Xiaolan Zhong, Yang Wang, Lingqian Chang, Fan Wang

**Affiliations:** 1 School of Physics, Beihang University, Beijing, China; 2 Key Laboratory of Biomechanics and Mechanobiology (Ministry of Education), Beijing Advanced Innovation Center for Biomedical Engineering, School of Biological Science and Medical Engineering, Beihang University, Beijing, China; 3 School of Biomedical Engineering, Anhui Medical University, Hefei, China; 4 School of Materials Science and Engineering, Beihang University, Beijing, China

**Keywords:** Optoelectronic devices and components, Photonic devices

## Abstract

Photoenergy harvesting is promising to power Internet-of-Things (IoT) sensors, freeing the limitations of wired power sources or batteries, enabling bio-integrated devices. However, existing photoenergy harvesting systems are restricted to solid or liquid configurations—limiting biocompatibility and space utilization—which makes obtaining flexible, biocompatible, light-harvesting devices a significant challenge. In this paper, inspired by natural ion gradient diffusion in organisms, offering an ion-hydrogel drops-based photoenergy harvesting base on ammonium molybdate. Through the photochemical process of ammonium molybdate, the electric potential of the device is regulated by the altered ion gradient and the redox pairs (${{[{\bf{Mo}}}_{{\bf{7}}}{{\bf{O}}}_{{\bf{24}}}]}^{{\boldsymbol{6}}-}$/${{[{\bf{Mo}}}_{{\bf{14}}}{{\bf{O}}}_{{\bf{46}}}]}^{{\boldsymbol{10}}-}$), to generate energy. When exposed to excitation light, a photo driven ammonium molybdate-hydrogel photoenergy harvester (PAPH) can generate an open-circuit potential of ~250 mV, and it can still obtain a considerable output power for milliseconds to thousands of seconds after the termination of the initial illumination. The reversible hydrogel droplets network allows for the recovery and fabrication of arbitrary structures of the PAPH. We further demonstrate the scalable PAPH networks can on-demand regulation of cell epithelial growth factor secretion and receptor expression, stimulate the cell proliferation, thereby facilitating biological tissue wound repair. This ionic hydrogel opens a new avenue for flexible, photoenergy harvesting, biocompatible devices.

## Abstract (web summary)

By using light-responsive molybdate-based ionic hydrogel droplets for photo energy harvesting, can generate a voltage output of ~250 mV.

## Graphical abstract

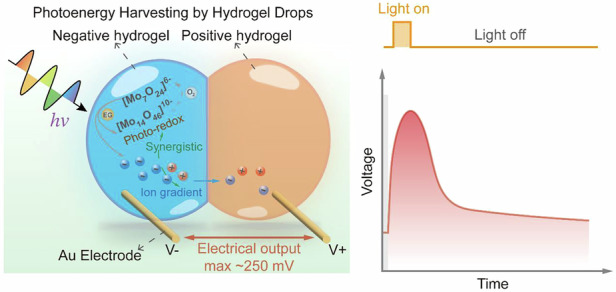

## Introduction

Energy harvesting technology offers a promising alternative to traditional continuous energy conversion and storage methods, aligning closely with the strategic goals of sustainable energy development. Optical power networks that harvest ambient light can power electronics^1,2^, soft robotics, and smart Internet of Things (IoT) sensors—eliminating the need for wired connections or battery replacements^3–5^. Ideally, an energy harvesting device (EHD) should combine biocompatibility, mechanical flexibility, and efficient energy conversion and storage. Yet, current implementations are predominantly based on solid or liquid systems in which separate conversion and storage modules are integrated^6,7^. This integration not only restricts compatibility and space or light utilization efficiency but also drives up manufacturing, maintenance, and repair costs due to the complexity of combining multiple components. As a result, it is challenging to develop flexible, biocompatible EHDs suitable for diverse applications^8,9^.

**Fig. 1:**
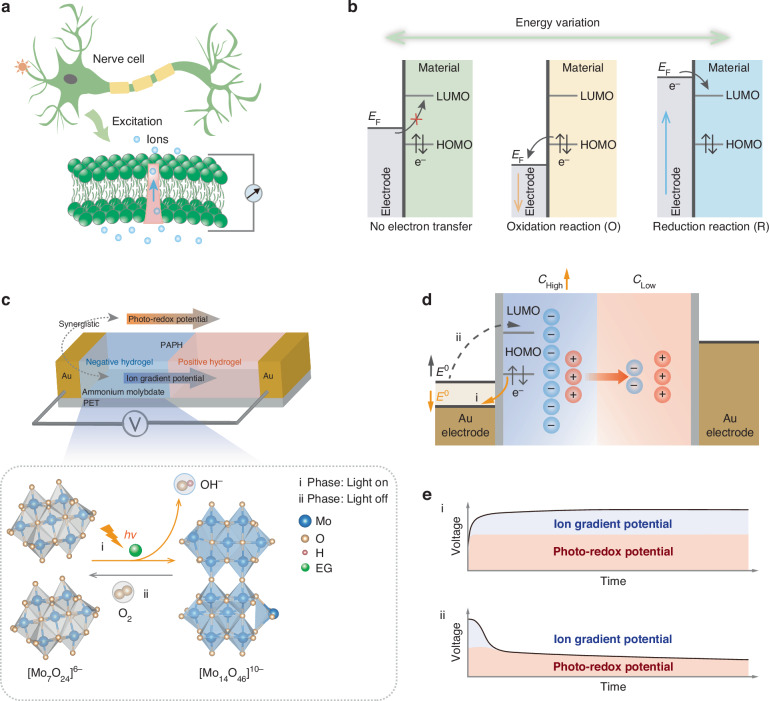
Structure and mechanism of PAPH. **a** The stimulated neuron cells cause the ion gradient inside and outside the cell membrane to change, generating an action potential. **b** Electron transfer and system energy change in redox process. **c** The structure and photochemical process change of the PAPH. Illustration: the photochemical process of ${{[{\rm{Mo}}}_{7}{{\rm{O}}}_{24}]}^{6-}$ and the oxidative recovery process of ${{[{\rm{Mo}}}_{14}{{\rm{O}}}_{46}]}^{10-}$. **d** Energy conversion mechanism of redox pair and ion gradient change. **e** Voltage change characteristics, light on process as show in (i) and light off process as show in (ii)

We drew inspiration from naturally evolving biological systems to address this issue. In biological systems, ion gradients^10,11^, or redox pairs, are extensively employed by organisms to transfer and utilize energy^12,13^, a process refined through natural selection, specifically the generation of action potential signals or photosynthesis within cells^10,14–17^. For the generation of action potential, stimulated neuron cells regulate the distribution of ions both inside and outside the cell membrane to form an ion hierarchical disparity, thereby establishing action potential signals (Fig. 1a). When neuron cells are stimulated and excited, it will cause the opening or closing of sodium ion (Na^+^) and potassium ion (K^+^) selective protein channels on the membrane so that it can be driven by the ion gradient one-way through, resulting in a potential difference ~100−150 mV^10,12,18–20^. Comparably, in chemical and physical systems, once a gradient is established, the system will spontaneously function in the direction of augmenting entropy to equilibrium the gradient until the system stabilizes, releasing energy during the process^21–24^. During photosynthesis, the energy from sunlight is absorbed by the photosynthetic pigments within chloroplasts, can convert light energy into chemical energy stored in adenine riboside triphosphate (ATP) and generates reducing coenzymes, which support subsequent energy conversion and utilization processes in biological systems^15,16,25^. This redox process can be harnessed to generate energy in artificial systems (Fig. 1b). During the redox reaction, electron transfer occurs, where oxidation lowers the material’s Fermi level, and reduction raises it. These shifts in the Fermi level result in changes in energy and can be reflected in the change of the system’s electrode potential. Thus far, researchers have proposed several biomimetic-based ion generators that generate electricity by constructing distinct ion gradients or harvesting environmental energy (such as light, heat, moisture, etc.) through the material into varying electrochemical potential energy gradients^7,12,18,25–29^. Nevertheless, due to the different energy generation mechanisms of ion gradient diffusion and redox reaction, their joint direct conversion of photoenergy harvesting devices has not been reported.

Ammonium molybdate is a recognized inorganic photochromic compound. In the presence of organic electron donors, ultraviolet illumination-induced excitation initiates photochemical process occurs, resulting in the gradient of negative ions (Supplementary Note 1). Importantly, the photoproduct during the process has a high reducibility (Offered by ${{[{\rm{Mo}}}_{14}{{\rm{O}}}_{46}]}^{10-}$) and long lifetime (Minutes or tens of minutes), depending on the oxidation activity in the environment. This time scale greatly increases the opportunity to utilize photoenergy, and it is a potential candidate to inhibit the recombination process of photogenerated charges. Based on this, we developed a photo driven ammonium molybdate-hydrogel photoenergy harvester (PAPH). The ions gradient and redox pairs (${{[{\rm{Mo}}}_{7}{{\rm{O}}}_{24}]}^{6-}$ and ${{[{\rm{Mo}}}_{14}{{\rm{O}}}_{46}]}^{10-}$) can be modulated by illumination, and the photo-responsive potential is produced by ions gradient and the electrochemical potential of redox pairs (I-PR). This mechanism, which has not been previously reported in existing inorganic photoelectric materials—largely based on the optoelectronic effect—can be utilized for both photoelectric conversion^9,25,30,31^. Unlike solid and liquid photoenergy harvesting systems, the hydrogel-based PAPH demonstrates superior flexibility, biocompatibility, low integration requirements, and ease of fabrication, making it highly promising for next-generation electronics. We illustrate that the output power of an expanded PAPH can be harnessed to regulate growth factor secretion and receptor expression in tissue epithelial cells, enabling on-demand photomanipulation of cell proliferation, betraying exciting potential in the field of bioelectronics.

## Results

### I-PR mechanism of the PAPH

The prototype of the PAPH (Fig. 1c) comprises two hydrogels droplets-based on gelatin, include a negative ammonium molybdate hydrogel droplet (n-gel) and a positive hydrogel droplet (p-gel) which absorbance spectrum is correspondingly exhibit photo responsive and photostable properties (Fig. S1and Fig. S2). They are deposited in sequence between gold electrodes on polyethylene terephthalate (PET) using a drip casting method (See Method). The droplets form a stabilized, support-free hydrogel structure within minutes, and SEM results of the completely dried samples revealed that the surface of the n-gel is rougher, which could be attributed to coupling with ammonium molybdate (Fig. S3). The wavelength of 365 nm light irradiation on the PAPH induces the photochemical process of molybdate ions coupled within the n-gel, generating negative ions of ${{[{\rm{Mo}}}_{14}{{\rm{O}}}_{46}]}^{10-}$ and ${{\rm{OH}}}^{-}$ as the photochemistry products. ${{[{\rm{Mo}}}_{7}{{\rm{O}}}_{24}]}^{6-}$ and ${{[{\rm{Mo}}}_{14}{{\rm{O}}}_{46}]}^{10-}$ constitute a reversible redox pair. As ${{[{\rm{Mo}}}_{14}{{\rm{O}}}_{46}]}^{10-}$ exhibits greater reducibility compared to ${{[{\rm{Mo}}}_{7}{{\rm{O}}}_{24}]}^{6-}$, the standard electrode potential delta $\triangle {E}^{0}=E\left({{[{\rm{Mo}}}_{14}{{\rm{O}}}_{46}]}^{10-}\right)-E({{[{\rm{Mo}}}_{7}{{\rm{O}}}_{24}]}^{6-}) < 0$, which reduces the standard potential of the counter electrode and resulted in photo-redox potential energy.

Meanwhile, the ions accumulation of ${{\rm{OH}}}^{-}$ on the n-gel side yields a modification in the ionic gradient, as anions migrate from high concentration (n-gel) to low concentration (p-gel), thereby releasing the ion gradient potential energy. The higher solvation entropy of ions leads to a steeper ion potential gradient. The potential generated by the ion gradient ${E}_{I}$ and the photo-redox pairs ${E}_{{PR}}$ both point from p-gel to n-gel (Fig. S4), the ions gradient and electrochemical potential of redox pairs (I-PR) results in an open-circuit voltage (${V}_{{\rm{oc}}}$) for the PAPH (Fig. 1d, ei). Using gold electrodes (Au) can generate electrical energy through the changes in ionic gradient potential and photo-redox potential induced by photoexcitation, which powers external electronic devices. This mechanism underpins our PAPH, which effectively unite redox pairs and ionic gradients within the system (Supplementary Note 1).

**Fig. 2:**
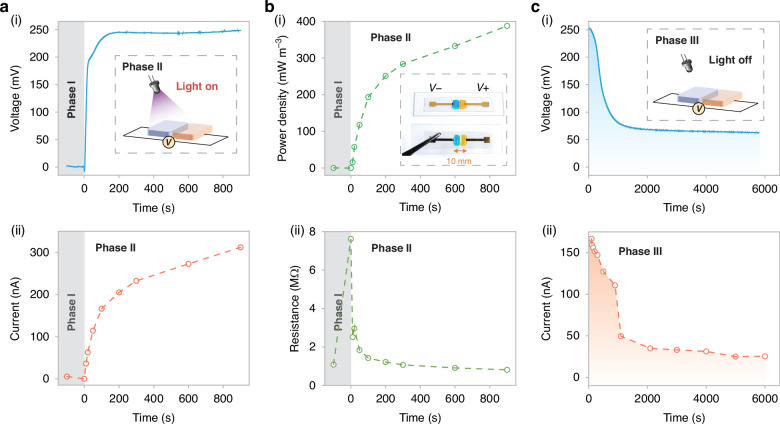
Output characteristics of the PAPH. **a**, **b** The output characteristics of PAPH when is excited (Wavelength of 365 nm, Power density of 9.9 mW cm^-2^): the normalized open circuit voltage (**a** (i)), short circuit current (**a** (ii)), output power (**b** (i)) and equivalent internal resistance (**b** (ii)). **c** The output characteristics of PAPH after removal of light field (Illumination for 100 s): open circuit voltage as show in (i) and short circuit current as show in (ii)

A key advantage of our PAPH is its ability to maintain open-circuit potential in the millivolt (mV) range for over an hour after the external light stimulus is removed (Fig. 2ci). This is significantly different from traditional photovoltaic cells. The decline in open-circuit voltage could be divided into two processes. In the initial process, the withdrawal of illumination would trigger the cessation of the photochemical procedure, thereby preventing the replenishment of ${{\rm{OH}}}^{-}$ on the n-gel side. Nonetheless, owing to the ionic gradient on either side, the ionic concentration on the n-gel side remained loftier than on the p-gel side before the diffusion equilibrium state. Thus, the spontaneous diffusion of ions would persist. With the unceasing augmentation of the ionic concentration on the p-gel side, a negative feedback regulatory effect of the electric field was created. Consequently, the rate of diffusion would progressively diminish until it dwindled to zero when the ionic concentrations on both sides attained equilibrium. The alteration of the ionic gradient assumed a leading role in the first stage. In the second process, the redox pair ${{[{\rm{Mo}}}_{7}{{\rm{O}}}_{24}]}^{6-}$ and ${{[{\rm{Mo}}}_{14}{{\rm{O}}}_{46}]}^{10-}$ are coupled with the n-gel, restricting its moved and resulting in an extended lifetime. Therefore, the PAPH could still uphold an open-circuit voltage of mV magnitude with illumination off. With the oxidation of ${{[{\rm{Mo}}}_{14}{{\rm{O}}}_{46}]}^{10-}$ by oxygen in environment, the open-circuit voltage would gradually diminish (Fig. 1d and Fig. 1eii).

### Output characteristics of the PAPH

Building on the proposed theory, analyze several key parameters of the PAPH. The electrical output characteristics of the single PAPH unit in the exposure and termination illumination as show in Fig. 2. When continuously illuminated by ultraviolet light (Wavelength of 365 nm, Power density of 9.9 mW cm^-2^), it generated ~250 mV at open-circuit voltage (${V}_{{\rm{oc}}}$) within 200 s and subsequently attains a plateau. Through theoretical deduction (Supplementary Note 1), the potential of PAPH can be expressed as follows:

1 $${E}_{{\rm{PAPH}}}=\left(-\frac{{S}_{R}{-S}_{O}}{{nF}}+{S}_{{\rm{td}}}\right)\,\cdot\, \Delta I$$

Where ${S}_{R}$ and ${S}_{O}$ correspondingly denote the entropy of the reduction and oxidation states of the system, n and ${F}$ represent the molar amount of electrons transferred implicated in the reaction and Faraday’s constant, ${S}_{{\rm{td}}}$ is the diffusion power under light induction, ∆*I* is the light intensity change of the applied light field.

In its nascent state, it exhibits rapid power growth under light excitation, with the fastest increase in voltage. With the consummation of the photochemical process, light response coefficient ${\alpha }_{{\rm{I}}}=\frac{{S}_{R}{-S}_{O}}{{nF}}$ will not undergo any alteration at this juncture, and the ${V}_{{\rm{oc}}}$ will remain invariable and not escalate. In contrast, when the PAPH is not excited, the ${V}_{{\rm{oc}}}$ gradually decreases from an extremely small initial background value due to charge equilibrium. This is in consonance with the regulation of ${V}_{{\rm{oc}}}$ alteration under continuous illumination in the experiment (Fig. 2ai and Fig. S5). The short-circuit current (${I}_{{\rm{sc}}}$) ascends rapidly to ~200 nA within 200 s, and continue to increase slowly and linearly as the elongation of illumination time. When ${V}_{{\rm{oc}}}$ reaches the plateau, it indicates that the system was in a dynamic equilibrium process. At that time, the ${{\rm{OH}}}^{-}$ continue to accumulate, when the short-circuit current is measured by shorting the PAPH, it would be forced to discharge instantaneously which like a capacitor, thus resulting in a linearly increasing relationship (Fig. 2aii). After 900 s of consecutive light activation, the output power density can reach ~387 mW m^-3^ (Fig. 2bi). The equivalent internal resistance increments when initially no light is applied, and commences to decrease when the light is applied to activate, which indirectly attests to the increase of the number of additional charged particles (Fig. 2bii). Through the changes of impedance spectrum and bode diagram before and after exposure to light, the device mainly exhibits double layer capacitance and diffusion control capacitance this is consistent with the previous explanation^32^. At middle and low frequencies, light excitation has a lower impedance, indicating an increase in diffused charged particles. In the low frequency, the phase Angle shows a linear trend with a high phase Angle, indicating that a capacitance-like controlled charge diffusion occurs. Further analysis of Raman spectra confirmed the asymmetric vibration of Mo-O, and the enhancement of 3100 cm^-1^-3600 cm^-1^ indicated the increase of ·OH and ·H (Fig. S6 and Fig. S7).

To better comprehend the PAPH operating principle, investigated the ${V}_{{\rm{oc}}}$ and ${I}_{{\rm{sc}}}$ behavior upon removal of illumination (Fig. 2c). A continuous illumination of 100 s was applied, and the ${V}_{{\rm{oc}}}$ reached ~250 mV, while the ${I}_{{\rm{sc}}}$ reached ~160 nA. After removing the illumination, due to the spontaneous movement of the gradient of charged particles in the system towards the equilibrium state, the ${V}_{{\rm{oc}}}$ gradually decreases over time from the initial voltage. Under the influence of system gradient entropy diffusion and the spontaneous oxidation process, the ${V}_{{\rm{oc}}}$ reduced. After 5000 s of deactivation, the ${V}_{{\rm{oc}}}$ remained ~75 mV. The ${I}_{{\rm{sc}}}$ was consumed at a significantly expedited rate during a period of 900 s and gradually dwindle to ~20 nA after 5000 s, which was occasioned by the air oxygen oxidation Mo (V) feeding back into the system. We measured the redox potential of the negative droplets before and after the photo-redox reduction reaction in the absence of a gelatin matrix, the reduction product would reduce the redox potential of the electrode, transforming it to -128.99 mV. The contributions of ionic diffusion calculated according to the theoretical limit and redox determined experimentally account for 36.1% and 50.1%, respectively. The influence of the gelatin matrix accounted for 13.8%. The error between the theoretical deduction value and the stable average value of the device was 4.43%, which verified the PAPH mechanism (Fig. S8). This result indicates that the voltage gain resulting from photoenergy harvesting is mainly composed of the ion gradient diffusion potential generated by molybdate ions during the photochemical process and the potential changes induced by variations in the redox couple.

**Fig. 3:**
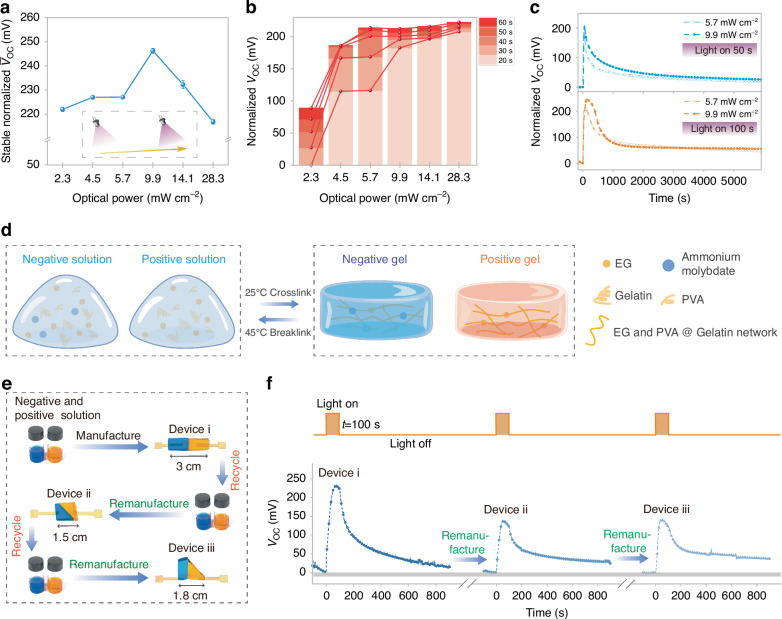
Output characteristics of the PAPH. **a**, **b** The steady-state open circuit voltage (${V}_{{\rm{oc}}}$) at different optical power levels (**a**) and the normalized open circuit voltage (${V}_{{\rm{oc}}}$) at different times (**b**). **c** When the optical power density is 5.7 mW cm^-2^ and 9.9 mW cm^-2^, the ${V}_{{\rm{oc}}}$ output curve of PAPH under different lighting times (50 s and 100 s). **d** Reversible transformation of droplet and hydrogel network for PAPH. **e**, **f** Reversible recovery and remanufacturing of PAPH (**e**) and output performance (**f**)

We further investigated the output characteristics of the PAPH under different light power levels (Power density *P*_d_ = 2.3 mW cm^-2^, 4.5 mW cm^-2^, 5.7 mW cm^-2^, 9.9 mW cm^-2^, 14.1 mW cm^-2^, 28.3 mW cm^-2^). As variations in light power can affect the photochemical processes of photoactive molybdate ions, leading to differences in ion gradients and redox couples, the PAPH final steady-state ${V}_{{\rm{oc}}}$ also exhibited variations (Fig. 3a and Fig. S9). When the optical power density is 9.9 mW cm^-2^, the PAPH exhibits the highest steady-state ${V}_{{\rm{oc}}}$ gain of ~250 mV. Analogous to our definition of light-induced diffusion power (Supplementary Note 1), we define the photo voltage power efficiency (${P}_{{\rm{eff}}}$) of PAPH which is used to evaluate the voltage gain efficiency under different optical power densities. The ${P}_{{\rm{eff}}}$ as follows:

2 $${P}_{{\rm{eff}}}=\frac{{\rm{Steady}}-{\rm{state}}\,{V}_{{\rm{oc}}}}{{\rm{Optical}}\,{P}_{{\rm{d}}}}\times 100\, \%$$

Compared to other optical power densities, when the *P*_d_ reaches 2.3 mW cm^-2^, the$\,{P}_{{\rm{eff}}}$ is greater, thus resulting the higher PAPH’s voltage gain efficiency. This indicates that the PAPH can generate considerable output even under lower excitation power, effectively harvesting and converting photoenergy. As optical power increases, the stable ${V}_{{\rm{oc}}}$ of PAPH initially rises but eventually declines, a behavior primarily driven by light diffusion. At lower power levels, with the increase of power, the change of photochemical reaction in the same time is more obvious, resulting in an increase the ${V}_{{\rm{oc}}}$. At higher power levels, accelerated photochemical reactions generate ions that are more efficient and diffuse more rapidly. This enhanced ion movement intensifies the negative feedback mechanism, ultimately reducing the ${V}_{{\rm{oc}}}$ (Supplementary Note 1). The ${V}_{{\rm{oc}}}$ change at different time points indicates that, before balance, the higher the optical power will cause the ${V}_{{\rm{oc}}}$ to rise faster (Fig. 3b). The time closer to where the light source is activated point, the ${V}_{{\rm{oc}}}$ variation trend is more obvious because the redox entropy change rate greater. The I-PR mechanism induces a saturation effect, causing the characteristic curve to asymptotically approach a plateau with rising excitation power levels. By comparing the ${V}_{{\rm{oc}}}$ of PAPH decay curve under illumination power density of 5.7 mW cm^-2^ and 9.9 mW cm^-2^, found that the decay potential after 3000 s minimally affected by power (Fig. 3c), as the impact of ion diffusion and redox entropy had become negligible by this point. The recycling and remanufacturing of optoelectronic devices serve as the foundation for achieving sustainable development, a process constrained by factors such as material characteristics, manufacturing processes, and recycling costs^33^. Benefit from the reversible gelatin network, the PAPH can achieve reversible conversion between the hydrogel network and the droplet within a temperature range of 25 °C and 45 °C (Fig. 3d). This feature endows the PAPH with flexible manufacturing and recycling capabilities, allowing for easy redissolution and reformation into different shapes (Fig. 3e). The output characteristics are shown in Fig. 3f, where some attenuation is observed after remanufacturing due to ion diffusion during the first discharge. After two cycles of recovery and remanufacturing, the voltage gain is still maintained at 61%. The observed discrepancy in output attenuation patterns between Fig. 3c and Fig. 3f following the removal of illumination might originate from device geometry, as shape or volume may influence the diffusion dynamics of ionic gradients or the recovery of redox pairs (Fig. S10).

### Scalable the PAPH networks

**Fig. 4:**
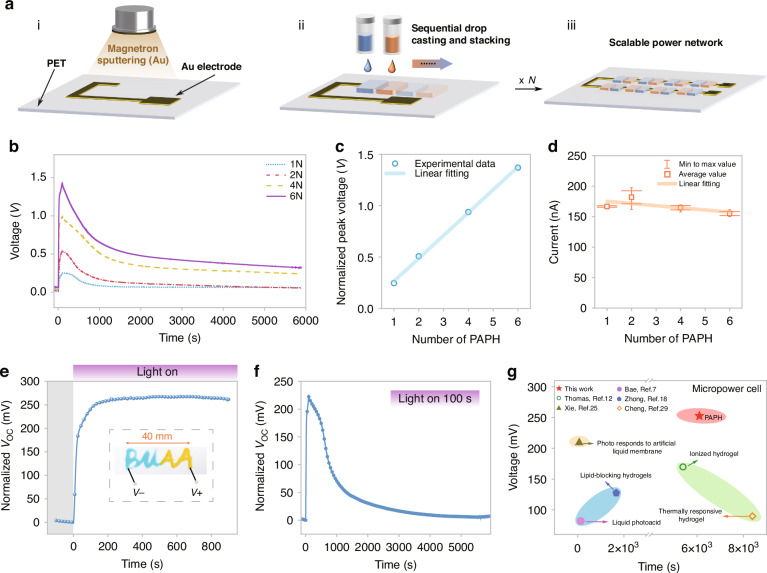
Scalable the PAPH networks. **a** Schematic diagram of the scalable networks by hydrogel droplet: electrode network preparation (i), droplet casting deposition (ii) and the PAPH expansion network (iii). **b**−**d** The output characteristics of different number in series (Optical power of 9.9 mW cm^-2^ and illumination for 100 s): open circuit voltage variation as show in (**b**); the normalized peak voltage gain shows a linear fitting law with the series unit (**c**) and the statistical short-circuit current (*n* = 3) average value (After illumination for 100 s) of different series units also approximately shows a linear law with the number of units (d). **e**, **f** The output performance of the “BUAA” PAPH which is described by hydrogel droplet ink (Optical power of 9.9 mW cm^-2^): the open circuit voltage (${V}_{{\rm{oc}}}$) curve of continuous illumination (**e**) and excitation for 100 s (**f**). **g** Performance comparison with other ion-gradient micro power cells, including the open circuit voltage and the time taken for the output voltage to decay by 75% after removal of the stimulus or circuit conduction

Since the PAPH’s ${V}_{{\rm{oc}}}$ generally remains unchanged with reduced size, the total output ${V}_{{\rm{oc}}}$ can be enhanced by increasing the number of series-connected smaller units. This approach is advantageous for expanding the application of hydrogel droplet assemblies. The electrode arrays were fabricated via a templated manufacturing approach, employing sequential droplet casting techniques to achieve configurations of 1, 2, 4, and 6 series-connected PAPH networks. In the experiment, the positive and negative hydrogels were alternately distributed in each array, and automatically aggregated and stabilized within several minutes, as shown in Fig. 4a. The output characteristics of the extended PAPH network are shown in the Fig. 4b–d (Optical power density of 9.9 mW cm^-2^ and illumination for 100 s). After 100 s of photoenergy harvesting, the peak voltages of different series-connected units exhibited a linear relationship. While the average short circuit current displayed a slight linear decay, which may be attributed to the increased impedance caused by series-connected electrodes. This suggests that we could achieve higher outputs through such network scaling approaches. When the series-connected units of *N* = 6, photoenergy harvesting induces a ${V}_{{\rm{oc}}}$ gain of ~1.4 V and ${I}_{{\rm{sc}}}$ of ~150 nA. After the light is turned off, the output characteristics can be decomposed into a single exponential combined with a Gaussian model, where are respectively represented ion diffusion action and photoredox pairs action (Fig. S11), further confirming that the voltage results from the interaction between ion gradient and redox pair. With the time interval of *T* = 200 s, by intermittently activating and deactivating the microgrid, the ${V}_{{\rm{oc}}}$ remained ~1.1 V after 50 cycles due to the diffusion of charged particles, which was still greater than 60% of the initial value (Fig. S12). To further showcase the flexible design capabilities of the PAPH, we created the ‘BUAA’ pattern using both positive and negative hydrogel materials. This pattern exhibited nearly identical open-circuit voltage characteristics under 9.9 mW cm^-2^ of light power excitation and achieved a stable voltage gain of ~250 mV (Fig. 4e, f). We compared the performance of the typical PAPH unit with other reported ion-gradient micro power cells^7,12,18,25,29^, including the open-circuit voltage and the time taken for the output voltage to decay by 75% after removal of the stimulus or circuit conduction (Fig. 4g). Our as-fabricated I-PR device ${V}_{{\rm{oc}}}$ was two to three times greater than some previously reported devices and the time taken for the output voltage to decay by 75% was one orders of magnitude higher than other devices. This demonstrates that our designed the PAPH can maintain higher voltage output over a longer duration.

### The PAPH accelerates cellular proliferation towards wound healing

**Fig. 5:**
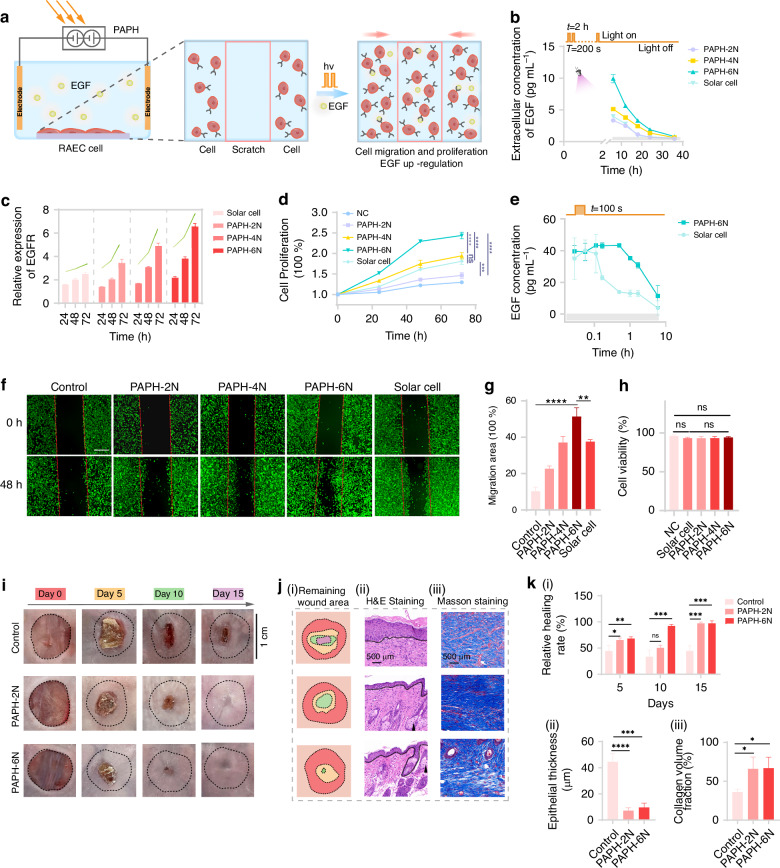
The effects of the electrical impulses generated by PAPH on cell behavior and epidermal tissue wound repair of mouse organism. **a** The photo-electrical effects generated by PAPH stimulate cells to release EGF and promote cell proliferation and migration. **b** ELISA was used to compare the trends in extracellular EGF concentrations secreted by the five groups of cells after a squire wave illumination (The period is *T* = 200 s, last for the first 2 h). The gray area in the figure represents the EGF concentration in the cell culture medium of the non-intervened control group at the same time point. **c** By using qPCR to quantify the amount of EGFR mRNA in the four experimental groups. Relative expression is calculated based on 2^−ΔΔCt^. The reference gene is GAPDH. d, Cell proliferation detected by CCK-8 assay. **e** ELISA was used to compare the trends in extracellular EGF concentrations secreted by cells after a single 100 s illumination, labeled by the orange area. **f** Representative images of RAEC cell migration; The red line represents the boundary of the scratch; scale bar, 200 µm. **g** Migration area of RAEC cells treated with PAPH (*N* = 2, 4, 6) and solar cell. The data indicate mean ± standard deviation (SD) from three experiments. ns, no significant difference. **P-value < 0.01. ***P-value < 0.001. ****P-value < 0.0001. **h** Quantitative comparison of cell viability after 3 times for different treatment groups. i, Representative digital images of wound areas treated with the PAPH (*N* = 2, 6 and T = 200 s for 2 h/day) on days 0, 5, 10, and 15. j, Stacked images of remaining wound area as show in (i), H&E staining of wound tissue (the skin spinous layer indicated by the black dashed line and sebaceous glands marked by black arrows) as show in (ii), Masson staining as show in (iii). **k** The healed area percentage of each treatment group of control and PAPH (*N* = 2, 6) as show in (i), Quantitative analysis of the H&E results revealed the epithelial thickness as show in (ii), Base on the Masson quantitative the collagen volume fraction as shown in (iii)

We further validated the value of the PAPH in biomedical applications. Wound healing remains a wide research field in regenerative medicine, which fundamentally rely on cellular proliferation. Enhancing the exocytosis of epithelial growth factor (EGF) is crucial for accelerating wound healing. Therefore, current efforts are taken upon technical innovation to enhance cell proliferation by forcing EGF secretion^34^. As the PAPH could provide electrical stimulation through photoelectric conversion, we employed this technology to accelerate wound healing by enhancing the efficiency of EGF secretion^35,36^. To assess PAPH’s ability to effectively promote EGF secretion, we established an in vitro model using rat aortic endothelial cells (RAEC), commonly used in wound healing research. EGF as a vital growth factor, promotes RAEC proliferation by binding to its specific receptor (Epithelial growth factor receptor, EGFR) on the cell surface^37^, activating downstream signaling pathways essential for cell proliferation and tissue regeneration (Fig. 5a). We examined the effect of PAPH networks (*N* = 2, 4, 6) and commercial photovoltaic arrays (~1.5 V) on extracellular EGF concentration over time in a periodic light field (*T* = 200 s for 2 h). PAPH network output differed from a square wave, with *N* = 6 resembling the open circuit potential of commercial photovoltaics (Fig. S12 and Fig. S13). The results indicated that the extracellular EGF concentration increased with tandem cells, demonstrating PAPH significantly enhances EGF secretion. When the PAPH series connections reached 6 N, EGF levels increased over 6-fold, 2.5 times higher than traditional solar cells. After the light exposure ended, EGF concentrations gradually decreased and returned to baseline (Gray area) within 24 hours (Fig. 5b). To maintain EGF expression and accelerate cell proliferation, a periodic light field (*T* = 200 s for 2 h/day) was applied. EGFR expression was assessed alongside EGF levels to verify EGF/EGFR pathway activation^35,36^. The PAPH can consistently elevated of EGF expression. And unlike commercial photovoltaics, which lose output power after light exposure, PAPH maintains output, significantly increasing EGFR expression (Fig. 5c). Compared to photovoltaic arrays with equivalent output power, the PAPH network (*N* = 6) doubled cell count in 48 hours, significantly outperforming commercial photovoltaics (Fig. 5d). Due to the extended I-PR change duration in the PAPH network (Fig. S11), short-time illumination (100 s) significantly enhancing EGF concentration. The PAPH (*N* = 6) had higher EGF concentration than commercial photovoltaic (PV) array, remaining twice as high even after 6 hours (Fig. 5e). Cell scratch assays further confirm enhanced cell migration due to EGF secretion (Fig. 5f, g). After 48 hours, wound healing percentages were ~10.3% (control), ~37.6% (PV array), and ~51.4% (PAPH-6N). The PAPH-6N achieved about 5 times more wound closure than the control group. Importantly, neither the PAPH nor the PV array affected cell viability, which remained >90%, comparable to the control group (Fig. 5h). These results suggest PAPH network more effectively promotes EGFR expression and EGF secretion through periodic light field excitation, enhancing EGF/EGFR binding and accelerating cell migration and proliferation by activating the EGFR pathway.

A mouse full-thickness skin wound model (BALB/c) with a 10 × 10 mm defect was established to evaluate PAPH’s therapeutic effect. PAPH networks (*N* = 2 or 6, *T* = 200 s for 2 h/day) were compared with conventional treatment (Control: gauze). Bio-medicine results showed the PAPH networks significantly promoted wound healing. The PAPH group exhibited smaller wound areas at all time points post-treatment (Fig. 5i). Stacked area images and statistical results also demonstrated PAPH significantly accelerated full-thickness wound healing. Healing rates in the first 5 days were ~20% and ~23% faster than control groups. Especially in the PAPH (*N* = 6) group, healing was nearly complete by day 10 (Fig. 5ji and Fig. 5ki). H&E staining showed the PAPH group had better wound healing quality (Fig. 5jii and Fig. 5kii). The control group’s skin tissue was significantly thickened, while the PAPH group promoted normal cell differentiation and tissue remodeling, preventing excessive proliferation. The spinous layer was thin and uniform, with numerous skin appendages and sebaceous glands. Collagen volume fraction (Masson) was quantified using ImageJ, verifying dermal repair advance by collagen content analysis in granulation tissue, the PAPH group had the highest collagen fiber content (Fig. 5jiii and Fig. 5kiii). These results demonstrated that promoted cell’s EGFR expression and EGF secretion by PAPH enhanced the wound healing effect.

## Discussion

In conclusion, we present a novel design for hydrogel droplets photoenergy harvesting devices based on ion gradients diffusion and photo redox pairs, enabling devices that maintains substantial output power for over an hour after illumination ceases. In the process of energy conversion, photoenergy is first converted into chemical energy, and then into electrical energy through the capacitance-like storage mechanism of the electrode. This highlights the potential of ions as energy carriers for photoelectric conversion and storage. With the flexibility and biocompatibility of hydrogel droplets, we further demonstrated its potential as a biomedical device by enhancing cell proliferation and facilitating tissue wound repair with significantly shorter illumination periods compared to commercial solar cells. Furthermore, this device will could be adapted for powerless in vivo applications. One limitation is its reliance on UV light, which restricts penetration depth or may cause tissue cells damage. However, utilizing energy transfer in dye-sensitized semiconductors^38,39^, or coupling with up-conversion nanoparticles (UCNP) to convert near-infrared (NIR) light to ultraviolet (UV) light provides a promising solution for achieving visible or NIR-excitable hydrogel droplets^40,41^. Additionally, the current power and energy density of this device remain relatively low compared to conventional photovoltaic or battery devices, which limits the practical application scope of hydrogel droplets. In future research, output power could be improved by introducing active materials or ions complementary to the molybdate ions’ photochemical processes in positive hydrogel droplets. Furthermore, designing hydrogel droplets with photochemically responsive battery or pseudocapacitive materials may also represent a potential optimization strategy^42,43^. In summary, this flexible, battery-free, and biocompatible hydrogel droplet photoenergy harvesting device offers a novel approach for regulating the activity of miniaturized cellular structures, it holds promise for powering in vivo microrobots, supporting tissue repair, and controlling next-generation bioelectronic hybrid interfaces^35,36,44^.

## Methods

### Prepare the hydrogel materials

All materials are purchased from Macklin and Yuanye Bio-Technology. In order to prepare the hydrogel photoenergy harvester, the positive and negative hydrogel materials are prepared by using reversible gelatin (Type A, Yuanye Bio-Technology) with low gelation and low dissolution temperature as hydrogel scaffolds. In brief, preparation the positive hydrogel material: 0.5 g polyvinyl alcohol (PVA, Molecular Weight: *M* = 195000, Macklin) is heated at 90 °C and dissolved into 10 g deionized water, followed by 1.5 g gelatin (Type A, Yuanye Bio-Technology) and 5 g ethylene glycol (EG, AR: 99%, Macklin), dissolved at 45 °C and used ultrasound 15 min to remove bubbles. Similarly, preparation the negative hydrogel material: 0.5 g polyvinyl alcohol (PVA, Molecular Weight: *M* = 195000, Macklin) is heated at 90 °C and dissolved into 10 g deionized water, then 0.5 g ammonium molybdate (Tetrahydrate, AR: 98%, Macklin), 1.5 g gelatin (Type A, Yuanye Bio-Technology) and 5 g ethylene glycol (EG, AR: 99%, Macklin), dissolved at 45 °C and used ultrasound 15 min to remove bubbles. The positive and negative hydrogel materials dyed with food coloring are only used for conceptual photography and non-testing purposes.

### Prepare photoenergy harvesting device

The photoenergy harvesting device is prepared by drip casting method. Before preparation, the positive and negative hydrogel materials were dissolved in a water bath at 45 °C, and then drip-cast on the electrode using a micro-syringe respectively, and then re-solidified at room temperature with different drip-cast patterns. Unless otherwise specified, the dimensions of the concept prototype are ~10 mm × 10 mm × 2 mm (positive hydrogel material and negative hydrogel material are ~10 mm × 5 mm × 2 mm, respectively). Electrode preparation: without special instructions, the gold electrode prepared on polyethylene terephthalate (PET) by magnetron sputtering is used for drip casting electrodes, which in general, argon gas was used to adjust the working pressure to 0.2 Pa and the sputtering time to 8 min with 5 W. The copper and zinc electrodes are derived from commercially available conductive electrodes. Similarly, scalable electrode networks are prepared using lithography methods. The coated (3000 rpm/min, 30 s) PET substrate with photoresist was lithographed using a mask plate (exposure wavelength 365 nm, exposure time 7 s). After 30 s of development, the gold deposited for 11 min by sputtering instrument. Finally, after removing the photoresist, clean it with deionized water for 3 times, dry and set aside.

### Characteristics of photoenergy harvesting device

We used electrochemical workstation (CHI660E, ChenHua) to record the electrical characteristics of the device and effectively evaluate the output characteristics. Unless otherwise specified, all specimens were tested 15 minutes after drop-casting completion, with excitation light fields applied at 100 seconds post-test initiation. We used an optical power meter to measure the power of the vertical incident photo field. Unless otherwise specified, the optical power is 9.9 mW cm^-2^ (Wavelength of 365 nm). The microstructure of freeze-dried samples was characterized using Scanning Electron Microscopy (SEM, S8000G, TESCAN). Spectral characterization: Raman spectroscopy (Raman spectra (DXR xi, Thermo) were performed under 532 nm laser excitation at room temperature), and ultraviolet spectrophotometer (TU1901, PERSEE).

### Experimental data processing

The output voltage was normalized to eliminate background discrepancies arising from friction, contact, and adsorption effects, enabling the analysis of light harvesting-induced variations in voltage gain. The *Normalized* *voltage* as follows:

3 $${Normalized\; voltage}=V-{V}_{{{\rm{I}}}_{0}}$$

Where V is the collected experimental data, and ${V}_{{{\rm{I}}}_{0}}$ is the voltage value when the excitation light field was initially applied.

Under continuous optical excitation at different optical power densities, the average output voltage of PAPH at steady state is taken as the Steady-state normalized voltage. The *Steady-state* *normalized* *voltage* as follows:

4 $$\,{Steady}-{state\; normalized\; voltage}=\frac{\sum {V}_{{\rm{Nor}}}}{{\rm{n}}}$$

Where ${V}_{{\rm{Nor}}}$ is the normalized voltage after steady state, and the sampling method involved selecting the steady-state curve segment after 600 s, with samples collected at 30 second intervals (*n* = 10).

### Cell culture and safety assessment

RAEC cells were grown in DMEM medium (Gibco) supplemented with 10% fetal bovine serum (FBS) and 1% penicillin/streptomycin (Gibco). The cells were incubated in a humidified incubator with 5% CO_2_ at 37 °C. The PAPH and solar cell arrays were connected to the cell culture dish using wires. Subsequently, the cells were irradiated with UV light for *T* = 200 s cycles, generating electrical stimulation for 2 hours per day over a period of 3 days. At the end of the 3 days, the cells were stained with Calcein-AM (Green) and propidium iodide (PI, Red). Fluorescence imaging was performed using a fluorescence microscope (IX83, Olympus). ImageJ (v.1.48) was used to count live cells (Green) and dead cells (Red) and to calculate cell viability.

### Cell proliferation experiment and scratch test

RAEC cells were cultured in a 12-well plate for 24 h (5 × 10^4^ cells/well). The wells were randomly divided into 5 groups: control group, PAPH-2N group, PAPH-4N group, PAPH-6N group, and solar cell group. The control group received no intervention. Here, the Solar cell group output power matched that of the PAPH-6N group, with a voltage of ~1.5 V and a current of ~160 nA. Cell proliferation of RAEC cells was assessed using CCK-8 assay (Gene-Protein Link, China). Absorbance was measured at 450 nm at 2 h, 24 h, 48 h, and 72 h using a Spark® multimode microplate reader (SPARK, TECAN). Cell viability was calculated using the following formula:

5 $${Cell\; proliferation\; rate}( \% )=[({{\rm{A}}}_{s}-{{\rm{A}}}_{0})\,({{\rm{A}}}_{b}-{{\rm{A}}}_{0})]\times 100\, \%$$

Where, ${{\rm{A}}}_{s}$ is the absorbance value of the experimental group (PAPH-2N group, PAPH-4N group, PAPH-6N group, and Solar cell group), including cells, cell culture medium, and CCK-8 solution. ${{\rm{A}}}_{0}$ is the absorbance value after cell adhesion at 2 hours post-seeding. ${{\rm{A}}}_{b}$ is the absorbance value of the control group, which includes non-intervened cells, cell culture medium, and CCK-8 solution.

The RAEC cells were cultured in a 12-well cell plate (1 × 10^5^ cells/well) and divided into the same 5 groups. After that, a scratch about 400 μm wide was made on the RAEC cells that covered the 12-well cell plate using the pipette tip (200 μL). The cells are intervened under different conditions. Finally, stain the cells with Calcein-AM (green) and image them at 0 h, 24 h, 48 h, and 72 h using a fluorescence microscope. ImageJ (v.1.48) was used to measure surface areas of the wounds.

6 $${Migration\; area}( \% )=[({{\rm{A}}}_{{\rm{initial}}}{-{\rm{A}}}_{{\rm{endpoint}}})/{{\rm{A}}}_{{\rm{initial}}}]\times 100\, \%$$

Where, ${{\rm{A}}}_{{\rm{initial}}}$ is the area of the scratch at the initial time point. ${{\rm{A}}}_{{\rm{endpoint}}}$ is the area of the scratch at the endpoint of the experiment.

### ELISA assay

RAEC cells cultured in complete DMEM were divided into five groups, and treated with PAPH (2 N, 4 N, 6 N) and solar cell. After the one-time treatment, cell culture supernatant was collected every 6 hours (*n* = 3), and EGF concentration in the supernatant was measured using an EGF kit (Gene-Protein Link) to observe the EGF levels in the cell supernatant.

### Real-time PCR reaction

After intervention, cells were digested from the culture dish using 0.25% trypsin, then centrifuged at 1000 g for 3 minutes and resuspended in DMEM with 10% FBS. Total RNA was extracted from the cells using the FastPure Cell Total RNA Isolation Kit V2 (Vazyme, RC112-01), and its concentration and purity were quantified using NanoQuant (Thermo Scientific, Waltham, USA). The total RNA was then analyzed by RT-qPCR using the One Step TB Green® PrimeScript™ RT-PCR Kit (TaKaRa, RR066A) with the following primers (Shenggong Biology): forward: (5′-GAGCAGAGATGTGAGGAGTCG-3′; reverse: 5′-TGGTTGACCCCCATTCTTGAG-3′). The amplification was conducted under the following conditions: initial heat at 42 °C for 5 min, 95 °C for 10 s, 95 °C for 5 s and 60 °C for 34 s (40 cycle), and eventually 72 °C for 2 min. Real time PCR amplification was performed with Quant Gene 9600 (Bioer, China). Relative expression is calculated based on 2^−ΔΔCt^. The reference gene is GAPDH. The relative expression level is calculated using the following formula:

7 $${Relative\; Expression}={2}^{-\Delta \Delta {\rm{Ct}}}$$

Where, $\Delta {\rm{Ct}}$ is the difference between the ${\rm{Ct}}$ values of the target gene and the reference gene: $\Delta {\rm{Ct}}={\rm{Ct}}({\rm{target}})-{\rm{Ct}}({\rm{reference}})$,$\,\Delta \Delta {\rm{Ct}}$ is the difference between the$\,\Delta {\rm{Ct}}$ values of the experimental group and the control group: $\Delta \Delta {\rm{Ct}}=\Delta {\rm{Ct}}({\rm{sample}})-\Delta {\rm{Ct}}({\rm{control}})$.

### Animal

All animal procedures were approved by the Biological and Medical Ethics Committee of Beihang University. Adult (8−10 week-old) male wild-type BALB/c mice (Body weight 18–25 g) were used and kept on a 12 h light/dark cycle at 22 °C -25 °C with ad libitum access to food and water. The mice were housed in groups (3 per cage) under standard conditions. These animals were purchased from the Vital River Laboratory (Animal Technology) in Beijing. During the skin tissue repair experimentation, the PAPH (*N* = 2, 6) is integrated into an annular bracket where the central opening of the device aligns with the wound area, while the annular region covers and adheres to the surface of healthy tissue surrounding the wound (Supplementary Note 2). Medical adhesive was applied at the base to secure the device, preventing slippage or deformation during application, thereby ensuring stable positioning of electrode regions around the target area throughout use.

## Supplementary information

Supplementary Information for Photoenergy harvesting by ammonium molybdate soft hydrogel drops

## Data Availability

All study data are available in the Article and its Supplementary Information. Additional information can be obtained from the corresponding authors upon reasonable request.
