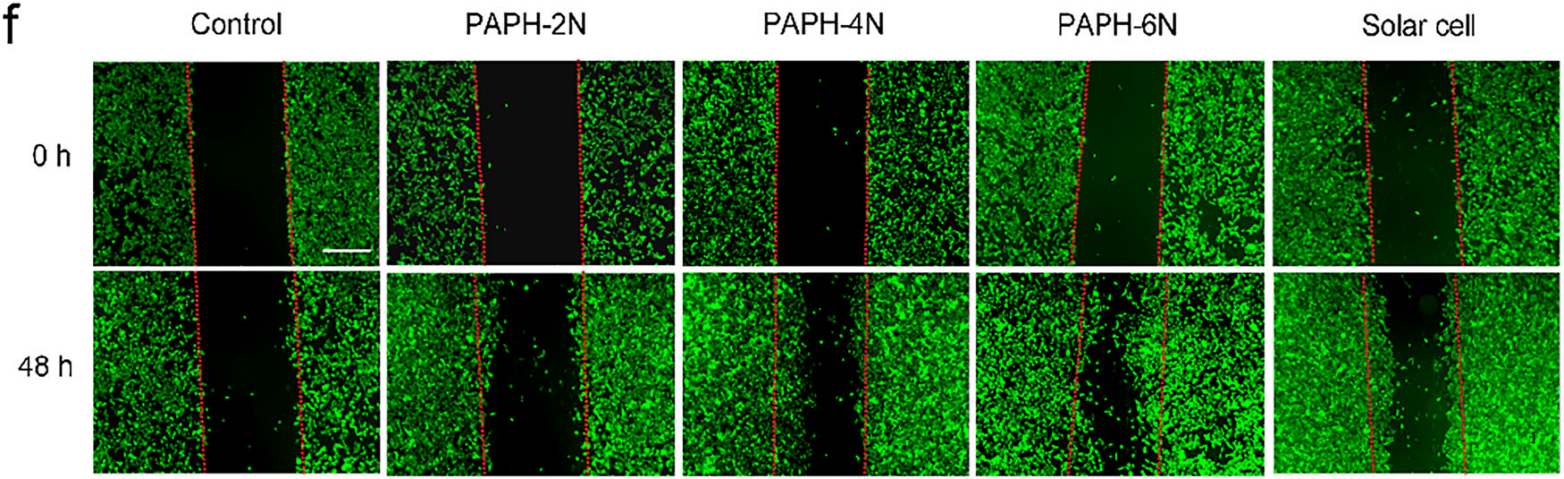# Correction: Photoenergy harvesting by ammonium molybdate soft hydrogel drops

**DOI:** 10.1038/s41377-026-02430-2

**Published:** 2026-08-04

**Authors:** Zelin Lu, Xinxin Hang, Zinan Zhao, Long Cheng, Yu Zeng, Bixuan Li, Menghan Tian, Baolei Liu, Xuchen Shan, Hongyan Zhu, Zhiying Wang, Menghao Ma, Jinliang Wang, Yongji Gong, Xiaolan Zhong, Yang Wang, Lingqian Chang, Fan Wang

**Affiliations:** 1 School of Physics, Beihang University, Beijing, China; 2 Key Laboratory of Biomechanics and Mechanobiology (Ministry of Education), Beijing Advanced Innovation Center for Biomedical Engineering, School of Biological Science and Medical Engineering, Beihang University, Beijing, China; 3 School of Biomedical Engineering, Anhui Medical University, Hefei, China; 4 School of Materials Science and Engineering, Beihang University, Beijing, China

**Keywords:** Optoelectronic devices and components, Photonic devices

Correction to: *Light: Science & Applications*10.1038/s41377-025-02016-4published online 21 October 2025

After publication of this article, it came to our attention that Figure 5f was incorrect and should be replaced. The PAPH-2N 0 h image was inadvertently duplicated in the Control 0 h panel, and the correct Control 0 h image was omitted. The figures are shown below. The original paper has been updated.

The incorrect Figure 5f:
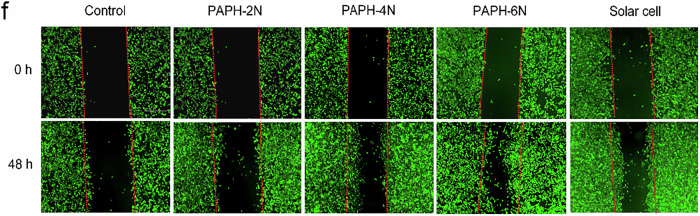


The correct Figure 5f: