# Supplementary material for: Photoenergy harvesting by ammonium molybdate soft hydrogel drops
Source: Light Sci Appl. 2025 Oct 21;14:372. doi: 10.1038/s41377-025-02016-4 (PMC12537955; doi:10.1038/s41377-025-02016-4)
Supplement: Supplementary file 1 — Supplementary Information for Photoenergy harvesting by ammonium molybdate soft hydrogel drops [file 41377_2025_2016_MOESM1_ESM.docx]

**Supplementary Information**

**Photoenergy harvesting by ammonium molybdate soft hydrogel drops**

**Zelin Lu^1^, Xinxin Hang^2^, Zinan Zhao^1^,** **Long Cheng^3^, Yu Zeng^2^, Bixuan Li^4^,** **Menghan Tian^1^,** **Baolei Liu^1^, Xuchen Shan^1^, Hongyan Zhu^1^, Zhiying Wang^2^, Menghao Ma^1^, Jinliang Wang^1^, Yongji Gong^4^, Xiaolan Zhong^1,^
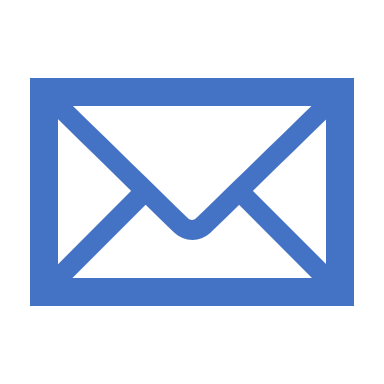
, Yang Wang^2,^
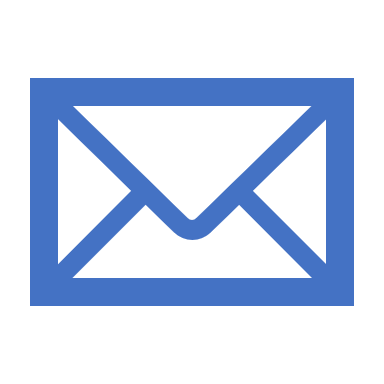
, Lingqian Chang^2,^
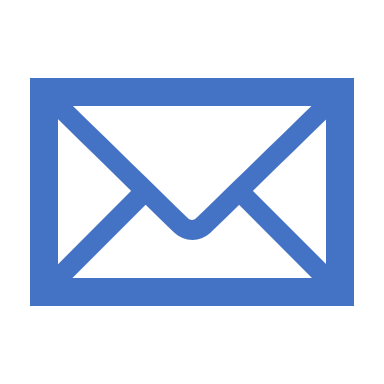
 & Fan Wang^1,^
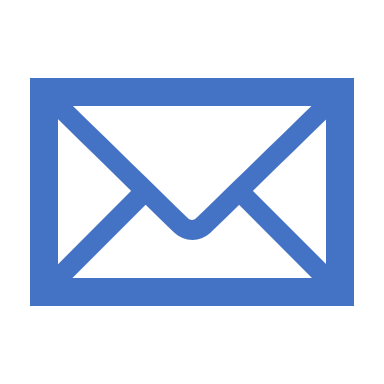
**

**Authors and Affiliations**

^1^ School of Physics, Beihang University, Beijing, China

^2^ Key Laboratory of Biomechanics and Mechanobiology (Ministry of Education), Beijing Advanced Innovation Center for Biomedical Engineering, School of Biological Science and Medical Engineering, Beihang University, Beijing, China

^3^ School of Biomedical Engineering, Anhui Medical University, Hefei, China

^4^ School of Materials Science and Engineering, Beihang University, Beijing, China

**Corresponding authors**

Correspondence to Xiaolan Zhong, Yang Wang, Lingqian Chang & Fan Wang

E-mail: [zhongxl@buaa.edu.cn](mailto:zhongxl@buaa.edu.cn); [wangyang2022@buaa.edu.cn](mailto:wangyang2022@buaa.edu.cn); [lingqianchang@buaa.edu.cn](mailto:lingqianchang@buaa.edu.cn); [fanwang@buaa.edu.cn](mailto:fanwang@buaa.edu.cn)

**Table of contents**

**Supplementary Note 1** ..................................................................................................................................... 2

**Supplementary Note 2** ..................................................................................................................................... 8

**Fig. S1** ............................................................................................................................................................. 10

**Fig. S2** ............................................................................................................................................................. 11

**Fig. S3** ............................................................................................................................................................. 12

**Fig. S4** ............................................................................................................................................................. 13

**Fig. S5** ............................................................................................................................................................. 14

**Fig. S6** ............................................................................................................................................................. 15

**Fig. S7** ............................................................................................................................................................. 16

**Fig. S8** ............................................................................................................................................................. 17

**Fig. S9** ............................................................................................................................................................. 18

**Fig. S10** ........................................................................................................................................................... 19

**Fig. S11** ........................................................................................................................................................... 20

**Fig. S12** ........................................................................................................................................................... 21

**Fig. S13** ........................................................................................................................................................... 22

**Fig. S14** ........................................................................................................................................................... 23

**Fig. S15** ........................................................................................................................................................... 24

**Reference** ....................................................................................................................................................... 25

**Supplementary Note 1. Theoretical background and** **mechanism derivation of design for photoenergy harvesting by ammonium molybdate soft hydrogel drops**

In brief, the mechanisms of the photo driven ammonium molybdate-hydrogel photoenergy harvester (PAPH) can be described in three parts.

**1.1 Part 1**

Polyoxometallic acid (Ammonium molybdate) is a common photochromic material. When there is an electron donor-organics such as alcohols, this material can undergo Mo (VI) to Mo (V) photo redox process under ultraviolet light (Wavelength of 365 nm) excitation. The active Mo (VI) involved ${{[Mo}_{7}O_{24}]}^{6-}$ in this process oxidizes the alcohols to produce hydroxide ($\mathrm{OH}^{-}$) and converts it into an unstable blue charged complex (${{[Mo}_{14}O_{46}]}^{10-}$), which is reductive and can be oxidized by an oxidizing agent to return to its initial charged state (Such as oxygen) ^1-3^. Therefore, ammonium molybdate with photochemical activity can change the charged particle gradient of the surrounding environment.

**1.2 Part 2**

Gelatin is a kind of translucent, high molecular weight polypeptide, usually derived from animal skin. It has low cost, well biocompatibility and biodegradability, and has been widely used in biology, medicine and other fields. There are abundant hydroxyl, carboxyl and amino groups in the molecular chain of gelatin, which makes it very easy to couple charged particles and functionalize ^4,5^. On the other hand, the non-covalent hydrogen bond in gelatin breaks reversibly at temperatures of about 40 °C, so at the right temperature, hydrogels using gelatin as a skeleton can achieve the conversion between liquid and colloidal ^6^. Therefore, we chose gelatin as the skeleton substrate. On the one hand, in the negative hydrogel droplet, the gelatin matched the appropriate amount of polyvinyl alcohol (PVA), it is coupled between the octahedral coordination structure of molybdate ions, where the larger-sized molybdate ions become structurally immobilized due to their coordination geometry constraints ^1,6-8^. Schematic representation of the chemical bond coupling between ammonium molybdate and matrix, as shown in **Schematic S1**. Therefore, these molybdate ions will not move which ensures the effective change of charged particle gradient in the negative hydrogel droplet. On the other hand, the conversion between liquid and colloid ensures that the PAPH can be assembled and constructed in any shape.


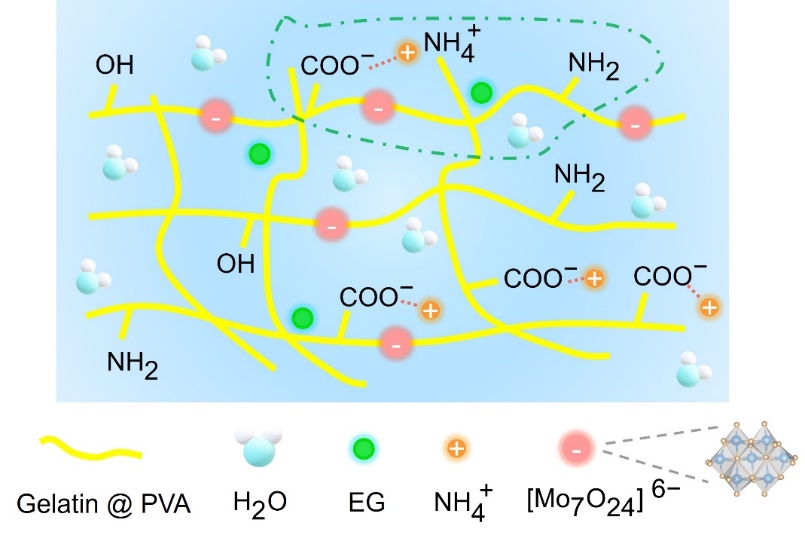


**Schematic S1 |** Chemical bond coupling diagram of ammonium molybdate and matrix.

**1.3 Part 3**

**Symbol definition of PAPH:**

The principle of PAPH is based on hydrogel droplets that can change the gradient of charged particles. When the PAPH is excited by light, the gradient of charged particles in the negative hydrogel droplet will occur accumulation. Due to effect of concentration diffusion, the changed charged particles in the negative hydrogel droplet will flow to the positive hydrogel droplet. This process will release the chemical energy and potential energy in the gradient of charged particles, and generate electromotive force between the electrode bridges. In fact, this process can be explained by the entropy transferred by the diffusion of charged particles caused by light excitation. Since the carrier that forms the current is the charged particle in the environment, this mechanism is different from the photoelectric effect (Photogenerated carriers) in the traditional sense. We define the light-induced diffusion power as the ratio of the electric field gradient to the intensity of the light field, which can be written as:

$S_{\mathrm{td}}=-\frac{\frac{dV}{dx}}{\frac{dI}{dx}}=-\frac{V\left( I_{\mathrm{open}} \right)-V(I_{0})}{I_{\mathrm{open}}-I_{0}}$ (S1)

In general, the light field intensity is 0 mW cm^-2^ when it is not excited. When the device is photoexcited, the PAPH is uniformly covered under the light field and the positive material does not participate in any photochemical process. Therefore, the photoinduced diffusion power of the PAPH can be written as:

$S_{\mathrm{td}}=\frac{V\left( I_{\mathrm{open}} \right)-V_{0}}{I_{\mathrm{open}}}$ (S2)

Where the $S_{\mathrm{td}}$is the photoinduced diffusion power, $V\left( I_{\mathrm{open}} \right)$ is the open circuit potential when the light field is opened. Light intensity$I_{\mathrm{open}}=\frac{P}{A}$, $P$ is the ultraviolet light power, $A$ is the action area. In simple terms, when the hydrogel droplet with photochemical reaction is defined as negative, the open circuit potential $V_{\mathrm{oc}}$ symbol is positive.

On the other hand, since only negative hydrogel droplets can undergo photochemical processes, the redox environment of the two electrodes before and after illumination is different, and the electrode potential at the interface of the two electrodes will change. Therefore, the final photo response potential includes not only the diffusion of charged particles caused by photo stimulated redox, but also the standard potential contribution of redox to the electrode. This will be explained further in subsequent derivations.

Because the general test environment is carried out at room temperature, the effect of temperature on the standard potential can be ignored here, so there is following:

$E|_{T}=E|_{T_{0}}-\frac{dE}{dT}\cdot\Delta\left（ T-T_{0} \right）=E|_{T_{0}}$ (S3)

That is, when the temperature is constant, the electrode potential changes can be approximated to be caused by redox pairs. Where, $E|_{T}$and $E|_{T_{0}}$ represent the standard potential at the two temperatures, and the change rate of electrode potential to temperature is the temperature coefficient $\alpha_{R}=\frac{dE}{dT}$.

Therefore, to sum up, the final potential change of the PAPH should be determined by the diffusion of charged particles caused by light induction and the redox potential change of the electrode. The mechanism is the change of charged particle gradient (${C [\mathrm{OH}]}^{-}$) and electrode potential caused by redox pairs changes（${{[Mo}_{7}O_{24}]}^{6-}$ and ${{[Mo}_{14}O_{46}]}^{10-}$）which be from photoexcitation of the negative hydrogel. Therefore, the open circuit potential output is defined as:

$\Delta E_{t}=\Delta E_{r}+\Delta E_{c}=\left( E_{r}-E_{r0} \right)+(E_{c}-E_{c0})$ (S4)

Where ${\Delta E}_{r}$represents the potential contribution change under the action of charged particles entropy, ${\Delta E}_{c}$represents the standard potential contribution change to the electrode under redox action. The combined action of $E_{r}\mathrm{and}E_{c}$ together constitutes the open circuit output potential $E_{t}$of the PAPH, which will be explained by step-by-step derivation.

1. **Potential contribution under the action of entropy of charged particles**

Under light induction, the photochemical processes of negative hydrogel droplets such as (**Part1**). Based on the Debye-Huckel equation ^9^, there is an exponential relationship between the activity coefficient of electrolyte and the reciprocal size, because the gelatin support coupling effect with ${{[Mo}_{7}O_{24}]}^{6-}$/ ${{[Mo}_{14}O_{46}]}^{10-}$ and the size are much larger than $\mathrm{OH}^{-}$. So, the contribution of ${{[Mo}_{7}O_{24}]}^{6-}$/${{[Mo}_{14}O_{46}]}^{10-}$ can be ignoring. According to the Nernst-Planck equation and the Goldman-Hodgkin-Katz current equation ^10,11^, the flux of ions $S$ in an electric field is given by $J_{S}$ (mol m^-2^ s^-1^), as follows:

$J_{S}=-D_{S}\left( \nabla C_{S}+\frac{FZ_{S}}{RT}C_{S}\nabla\varphi\right)$ (S5)

Where $D_{S}$ (m^2^ s^-1^) is the diffusion coefficient of $S$ in medium, $C_{S}$ (mol m^-3^) is molar concentration of ion $S$, $F$ is Faraday's constant, $Z_{S}$ is the charge of $S$, $R$ (J mol^-1^ K^-1^) is gas constant, $T$ (K) is the temperature and $\varphi$ (V) is the electric potential. The ion flux in current form, according to assumptions based on the Goldman-Hodgkin-Katz current equation ^10,11^, as follows:

$I_{S}=P_{S}VF^{2}Z_{S}^{2}\frac{C_{S_{\mathrm{in}}}-C_{S_{\mathrm{out}}}e^{-VF\frac{Z_{S}}{RT}}}{RT\left( 1-e^{-VF\frac{Z_{S}}{RT}} \right)}$ (S6)

Where $I_{S}$ (A m^-2^) is the current density caused by the change of ion $S$, $P_{S}$ (m s^-1^) is the permeability of ion $S$, $V$ (V) is the resulting electric potential, $F$ is Faraday's constant, $Z_{S}$ is the charge of $S$, $C_{S_{\mathrm{in}}}$ and $C_{S_{\mathrm{out}}}$ (mol m^-3^) is the ion $S$ concentration inside and outside the electrode when the photo responsive PAPH has an output, $R$ (J mol^-1^ K^-1^) is gas constant, $T$ (K) is the temperature. Here, since it is mentioned earlier that it can be approximated as only $\mathrm{OH}^{-}$at work, the above formula can be written as:

$I_{S}=\frac{VF^{2}P_{\mathrm{OH}^{-}}}{RT\left( 1-e^{\frac{VF}{RT}} \right)}\cdot\left[ C_{S_{\mathrm{in}}}-C_{S_{\mathrm{out}}}e^{\frac{VF}{RT}} \right]=\frac{VF^{2}P_{\mathrm{OH}^{-}}}{RT\left( 1-e^{\frac{VF}{RT}} \right)}\cdot\left[ C_{\mathrm{OH}^{-}\mathrm{in}}-C_{\mathrm{OH}^{-}\mathrm{out}}e^{\frac{VF}{RT}} \right]$ (S7)

Previously, we discussed the relationship between ion flux and the ideal electromotive force, and the voltage generation can be modeled according to the internal resistance, because the positive material is light stable, it accounts for a large proportion of the internal resistance. When the PAPH open circuit, the external resistance is much greater than the internal resistance ($R_{\mathrm{meter}}\gg R_{\mathrm{source}}$), as shown in **Schematic S2**. Therefore, the PAPH at this time will enter a state where the output voltage ($V_{\mathrm{oc}}$) is high and the current $I$→ 0, so we have:

$V_{\mathrm{oc}}|_{E_{r}}=\frac{RT}{F}\cdot\ln\frac{C_{\mathrm{OH}^{-}\mathrm{in}}}{C_{\mathrm{OH}^{-}\mathrm{out}}}=V_{\mathrm{meter}}$ (S8)

When short circuited，$R_{\mathrm{meter}}\to0\ll R_{\mathrm{source}}$, therefore, $V_{\mathrm{meter}}=0$. The short-circuit current ($I_{\mathrm{sc}}$) output can be written as:

$I_{\mathrm{sc}}=I_{\mathrm{meter}}=\frac{V_{\mathrm{oc}}}{R_{so\mathrm{urce}}}$ (S9)


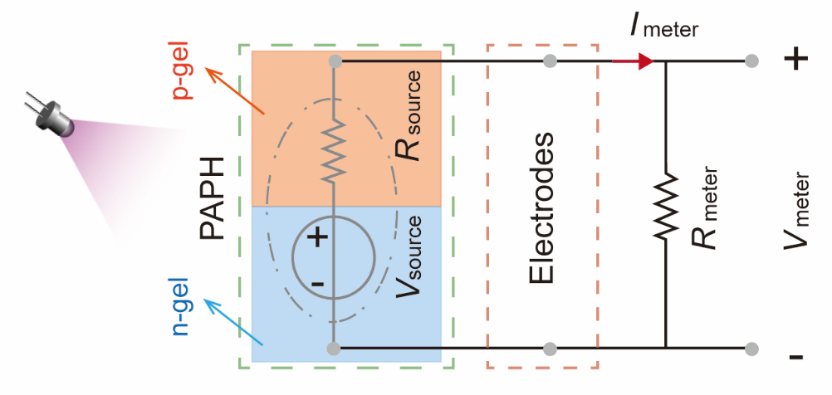


**Schematic S2 |** Equivalent circuit diagram of electrical measurement connection electrode and measuring instrument of optical response PAPH.

So, to sum up, we can know that for positive droplet materials, the additional ion concentration will hinder and neutralize the diffusion of $\mathrm{OH}^{-}$ (Negative droplet material), which will lead to decrease in the open circuit potential. As a design trade-off, we only use the charged particles in gelatin (Type A) to balance the effect of ammonium ions in the negative material, i.e.no additional ions are introduced into the positive material unless otherwise stated. The above expression (S8) shows the potential change caused by ion diffusion in the simplified case, but it cannot determine the rule under light condition. In the following, we will further analyze the rule of potential change caused by light induced ion diffusion when light on and off. To facilitate analysis, under thermodynamic equilibrium conditions, the photochemical equilibrium constant ($K$) in the photoinduced negative material is introduced. As the predominant variations arise from ionic changes in the system, the contributions of other species or products (Such as EG or its organic oxides) are negligible, therefore, the $K$ as follows:

$K=\frac{{{C^{0.5}[Mo}_{14}O_{46}]}^{10-}\cdot C[{OH]}^{-}}{C{{[Mo}_{7}O_{24}]}^{6-}}$ (S10)

Where, $C$ is the concentration or activity of reactants and photochemical products.

When temperature and pressure are constant, the free energy changes as follows:

$\Delta G=\Delta G^{0}+RT\cdot\ln K$ (S11)

Where, $\Delta G^{0}$ and $R$ are respectively express the Gibbs free energy change and the gas constant under the standard state, $T$ is the temperature. In electrochemistry, a potential on a cell electrode is defined as a reduction in the Gibbs free energy per unit of coulomb charge transfer, can be written by:

$\Delta G=-ZFE=-Z_{\mathrm{OH}^{-}}\cdot F\cdot E$ (S12)

Where, $Z$is the number of electrons transferred by the reaction, $F$ is Faraday's constant and $E$ is the potential. Here we temporarily do not discuss the effect of redox on the electrode interface potential, only consider the potential change caused by $\mathrm{OH}^{-}$ diffusion, so it can be written as:

$E_{r}=E^{0}-\frac{RT}{ZF}\cdot\ln K$ (S13)

Therefore, the change in potential due to a change in the gradient of charged particles can be written as:

${\Delta E}_{r}=E^{0}-\frac{RT}{ZF}\cdot\ln\Delta K$ (S14)

Where the $E^{0}$ is electrode potential under the standard state. Since there is no change in $\Delta K$ involved in the positive material, the open circuit potential change under light excitation as follows:

${\Delta V}_{r}={\Delta E}_{r}|_{\mathrm{positive}}-{\Delta E}_{r}|_{\mathrm{negative}}=\frac{RT}{ZF}\cdot\ln\Delta K=\frac{RT}{ZF}\cdot\ln\frac{{\Delta C}^{0.5}{{[Mo}_{14}O_{46}]}^{10-}\cdot{\Delta C[OH]}^{-}}{\Delta C{{[Mo}_{7}O_{24}]}^{6-}}$ (S15)

Since the previously mentioned contribution is small of ${{[Mo}_{7}O_{24}]}^{6-}$/${{[Mo}_{14}O_{46}]}^{10-}$ that is coupled with hydrogel matrix chemical chains, therefore, the above expression can be approximated as follows:

${\Delta V}_{r}=\frac{RT}{F}\cdot\ln\Delta C{(OH}^{-})$ (S16)

Therefore, in the light excitation state, the potential change limitation caused by the change of the gradient of charged particles induced by light induction is as follows:

$V_{r}|_{\mathrm{limit}}=\frac{RT}{F}\cdot\ln\Delta C{(OH}^{-})=-\frac{RT}{F}\cdot\ln C{(OH}^{-})|_{\mathrm{produce}}$ (S17)

When the light is removed, referring to the previous formula (S8), we assume that the $\mathrm{OH}^{-}$ in the negative material when the light field is removed is $C{(OH}^{-})|_{I}$, which can be seen as a constant, so the output potential is:

$V_{r}=\frac{RT}{F}\cdot\ln\Delta C{(OH}^{-})=\frac{RT}{F}\cdot[\ln C{(OH}^{-})|_{I}-\ln C{(OH}^{-})|_{\mathrm{out}}]=\frac{RT}{F}\cdot\ln\frac{C{(OH}^{-})|_{I}}{C{(OH}^{-})|_{\mathrm{out}}}$ (S18)

Therefore, to sum up, under ideal conditions, the theoretical analysis of potential changes caused by diffusion is consistent with the experimental results. In fact, for the effect of diffusion, we should also consider the interaction between ion equilibrium and the effect of photochemical equilibrium, but this will only affect the relationship between output potential and time, but the overall trend is unchanged.

1. **Electrode potential contribution under redox action**

However, when redox reactions are involved in the system, the effect of diffusion cannot simply be considered ^8^. The redox reaction at the electrode interface (${{[Mo}_{7}O_{24}]}^{6-}$/${{[Mo}_{14}O_{46}]}^{10-}$) will affect the redox potential of the electrons in the electrode, in which case we need to consider the effect of redox reaction.

$O+ne\leftrightharpoons R$ (S19)

Where $O$ is the oxidized states and $R$ is the reduced states. In the PAPH samples, $O$ is ${{[Mo}_{7}O_{24}]}^{6-}$ and $R$ is ${{[Mo}_{14}O_{46}]}^{10-}$. At the equilibrium, the net change in electrochemical potential should be zero at the electrodes. Therefore, the changes in the electrochemical potential as follows:

$\left\{ \begin{aligned} \tilde{\mu}_{O}+n\tilde{\mu}_{e}=\tilde{\mu}_{R} Negative material electrode \\ 0 Positive material electrode \end{aligned} \right.$ (S20)

Where the substances $O$ and $R$ electrochemical potential expressed as$\tilde{\mu}_{i}= \mu_{i}+q_{i}V$ ($i$=$O$,$R$), including chemical potential part $\mu_{i}$ and electrostatic potential part $q_{i}V$. The $q_{i}$ is the charge of substance $i$, and $V$ is the electrostatic potential in the electrolyte. The electrochemical potential per mole of electrons is$\tilde{\mu}_{e}= E_{F}-FV_{e}$, $E_{F}$is the Fermi level of the electrode, $V_{e}$is the electrostatic potential in the electrode, and $F$ is the Faraday’s constant. Since the Fermi level in a metal electrode can be considered a constant, the electrochemical potential difference between the positive and negative materials is the electrode voltage difference caused by the photoexcited redox process:

$n\Delta\tilde{\mu}_{e}=\Delta\tilde{\mu}_{R}-\Delta\tilde{\mu}_{O}=\left( \Delta\mu_{R}-\Delta\mu_{O} \right)+q_{R}\Delta V-q_{O}\Delta V=\left( \frac{\partial\mu_{R}}{\partial I}-\frac{\partial\mu_{O}}{\partial I} \right)\cdot\Delta I+\left( q_{R}-q_{O} \right)\cdot\Delta V$

$=-\left( S_{R}{-S}_{O} \right)\cdot\Delta I+(-nF)\cdot(-S_{\mathrm{td}}\cdot\Delta I)$ (S21)

Where the $S_{\mathrm{td}}=-\frac{\Delta V}{\Delta I}$, $S_{i}=-\frac{\partial\mu_{i}}{\partial I}$ ($i$=$O$,$R$), since charge is conserved, for every mole of reaction, $q_{R}-q_{O}=-nF$. Therefore, the total power of the photoinduced voltage potential is:

$S=\frac{\Delta\tilde{\mu}_{e}}{nF\Delta I}=-\frac{{\Delta V}_{e}}{\Delta I}=-\frac{S_{R}{-S}_{O}}{nF}+S_{\mathrm{td}}$ (S22)

Here, we define the light response coefficient $\alpha_{I}=\frac{S_{R}{-S}_{O}}{nF}$, define the change in entropy of the redox reaction as $\Delta S_{pr}=S_{R}{-S}_{O}$. Therefore, the total photoinduced voltage potential power can be simplified as:

$S=S_{r}+S_{c}=S_{\mathrm{td}}-\alpha_{I}$ (S23)

So, combined with 1) and 2), the total voltage of PAPH can be expressed as:

$E_{\mathrm{PAPH}}=\left（ S_{\mathrm{td}}-\alpha_{I} \right）\cdot\Delta I=(-\frac{S_{R}{-S}_{O}}{nF}+S_{\mathrm{td}})\cdot\Delta I$ (S24)

Where, the $\Delta I$ is the light intensity change of applied light field, $n$ represent the molar amount of electrons transferred implicated in the reaction, and according to the previous definition $S_{\mathrm{td}}\cdot\Delta I=V_{r}$.

It can be observed that as the progress of the photochemical reaction, the voltage growth power is gradually reduced, and the initial state has the highest growth power under light excitation, that is, the voltage growth is the fastest. Under constant light, as the photochemical process is fully reacted, $\alpha_{I}$can then be regarded as a fixed value, and the open circuit potential will remain constant and not increase. This is consistent with the rule of open circuit potential under continuous illumination in the experiment.

Therefore, the power of the photoinduced voltage can be written as:

$S=\left\{ \begin{aligned} -\frac{S_{R}{-S}_{O}}{nF}+S_{\mathrm{td}}, Light on \\ -\frac{S_{O}{-S}_{R}}{nF}+S_{\mathrm{td}}, \mathrm{Lig}ht off \end{aligned} \right.$ (S25)

Where,

$S_{\mathrm{td}}=\left\{ \begin{aligned} -\frac{RT}{F\Delta I}\cdot\ln C{(OH}^{-})|_{\mathrm{produce}}, Light on \\ \frac{RT}{F\Delta I}\cdot\ln\frac{C{(OH}^{-})|_{I}}{C{(OH}^{-})|_{\mathrm{out}}}, Light off \end{aligned} \right.$ (S26)

Therefore, in summary, it is proved that the photoinduced voltage power of the PAPH is the result of the combined contribution of two different mechanisms (I-PR mechanism, The photoinduced charged particles diffusion and the electrode potential change is caused by redox pairs change which because of photochemical processes ${{[Mo}_{7}O_{24}]}^{6-}$/${{[Mo}_{14}O_{46}]}^{10-}$).

**Supplementary Note 2. Methods and mechanisms of PAPH to regulate cell proliferation and wound healing**

Bioelectricity is an indispensable part of the living system, in which the endogenous electric field plays a vital role in cell proliferation, differentiation, communication and transport ^12^. As a kind of non-drug physical stimulation, external field electrical stimulation can regulate the endogenous electric field of cells or living systems, enhance the expression of specific genes in cells, and improve cell behavior, such as proliferation and differentiation, and has been widely used in biomedicine ^13^. Because of these effects, electrical stimulation shows great potential in areas such as tissue repair, nerve stimulation, and bone regeneration. In recent years, various power systems have been designed as field sources, such as piezoelectric, photoelectric, ionized hydrogel batteries ^12-15^. In short, there are many encouraging examples, but our work proposed a light collection device for inorganic hydrogel droplets and demonstrates that the output power can as demand to promoting cell proliferation and migration. To further confirm, we also made a comparison of commercial PV system (**Schematic S3**) with the almost same power as the PAPH extended networks (N=6).


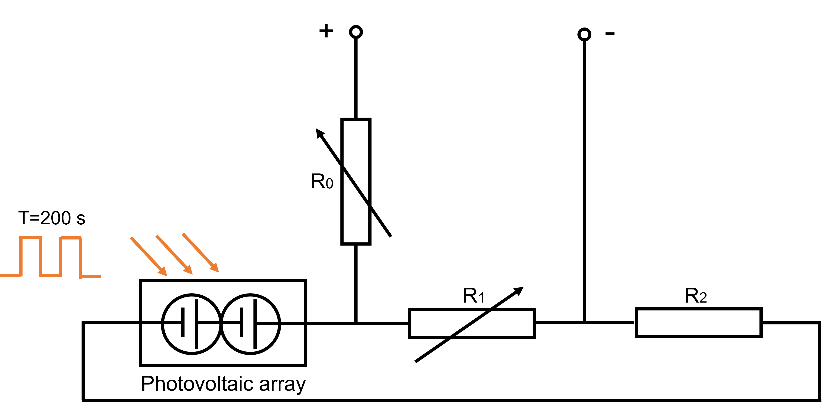


**Schematic S3 |** The commercial photovoltaic system circuit diagram which used in a control cell proliferation experiment to comparison with the PAPH extended networks.

During the skin tissue repair experimentation, we arranged the flexible hydrogel droplets PAPH (N=2, 6) within an annular bracket. Specifically, by employing the annular bracket structure ^16^, the central aperture of the device is aligned with the wound area, while the annular region covers and adheres to the healthy tissue surrounding the wound (**Schematic S4**). Owing to the hydrogel droplets' flexibility, biocompatibility, and self-adhesive properties, it is unnecessary to employ additional adhesives to construct the hydrogel droplet network within the device. The annular bracket provides external mechanical support for the hydrogel droplets. Furthermore, medical adhesive is applied to the dorsal side of the bracket to immobilize the device, preventing slippage or deformation during applying and ensuring consistent stabilization of the electrode array around the target region. And maintains reliable adhesion and stimulation efficacy even under conditions of skin dynamic stretching or organism movement.


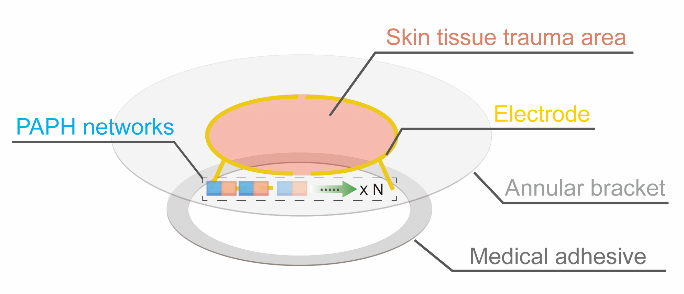


**Schematic S4 |** Schematic diagram of the annular bracket for tissue trauma repair.

**
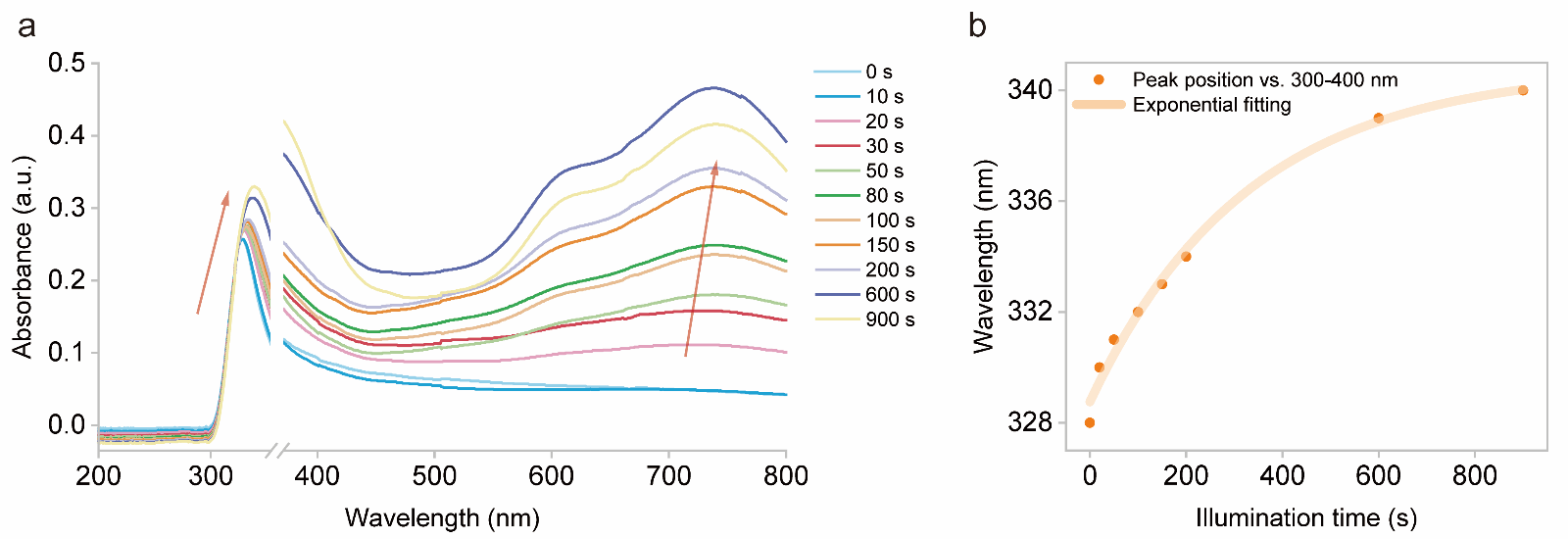
**

**Fig. S1 | a,** Absorbance spectra of negative hydrogel droplets at different photo excitation times (Wavelength of 365 nm, Power density of 9.9 mW cm^-2^). **b,** Displacement distance of absorption peak with time.


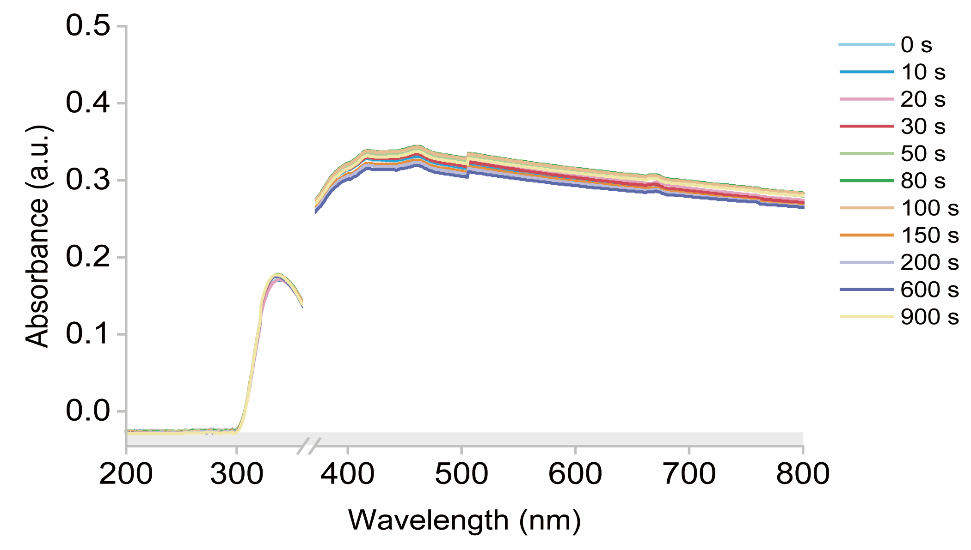


**Fig. S2 |** Absorbance spectra of positive hydrogel droplets at different photo excitation times (Wavelength of 365 nm, Power density of 9.9 mW cm^-2^).


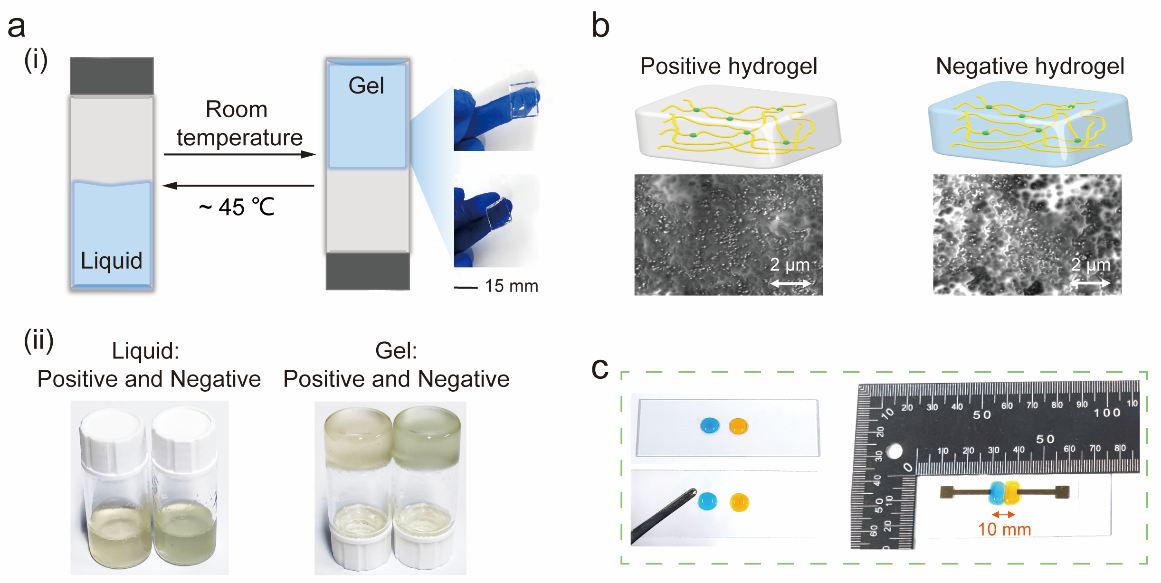


**Fig. S3 |** **The** **PAPH for flexibility and photos. a,** Flexible droplet ink can achieve reversible conversion of hydrogel and droplet between room temperature to ~45 ℃ (i) and actual photos (ii). **b,** SEM images of the dried positive and negative hydrogel samples. **c,** The PAPH prepared by n-gel droplets (Blue) and p-gel droplets (Orange). The hydrogel materials dyed with food coloring are only used for conceptual photography and non-testing purposes.

**
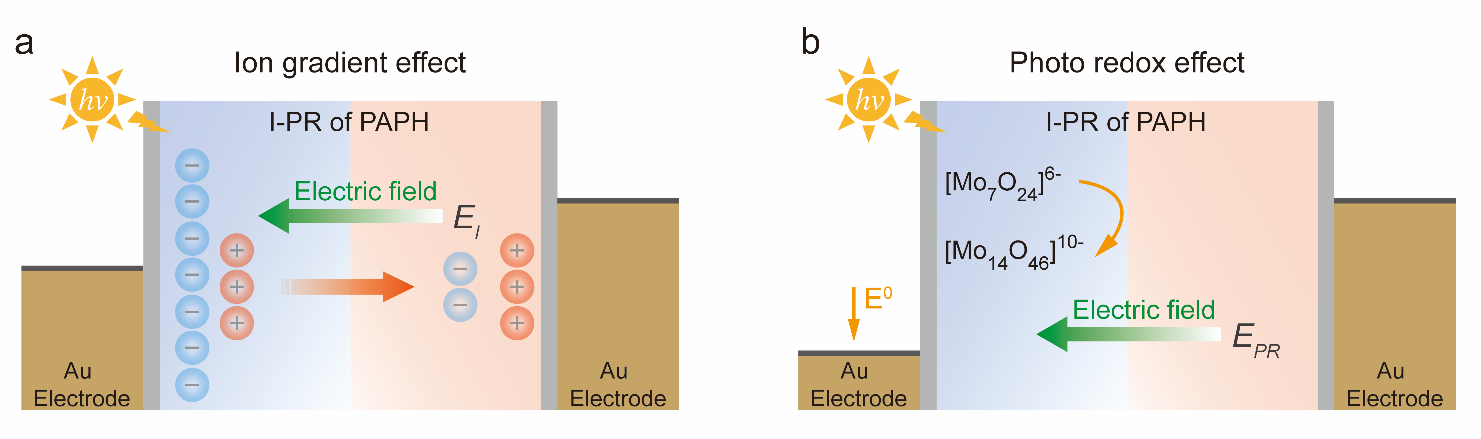
**

**Fig. S4 | The mechanism of generating electric field direction.** The direction of electric field caused by the change of ion gradient (a) and the change of photoinduced redox pair (b).


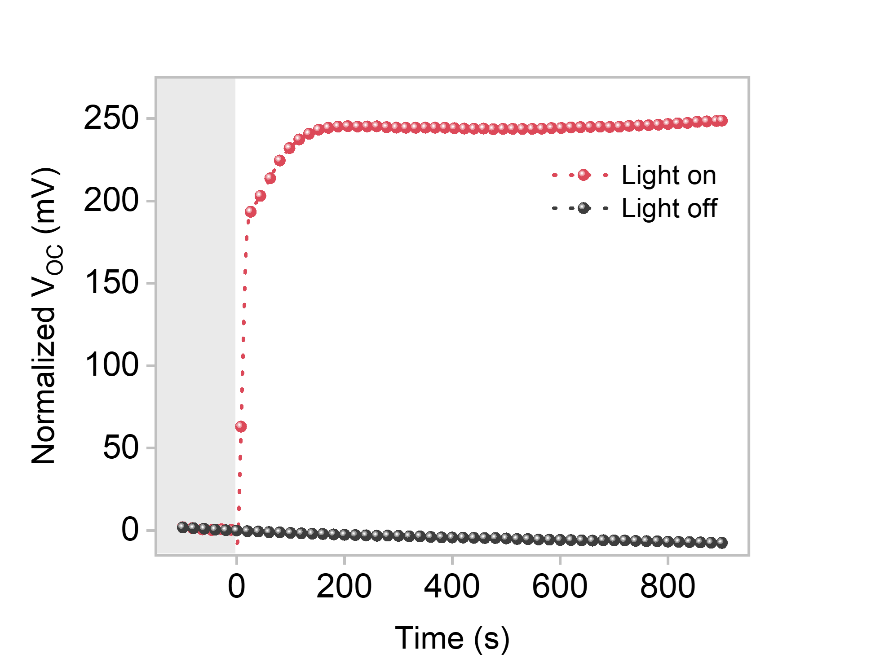


**Fig. S5 |** Comparison of open circuit voltage with dark state of the PAPH under continuous illumination (Applied at ‘0 s’, Wavelength of 365 nm, Power density of 9.9 mW cm^-2^).

**
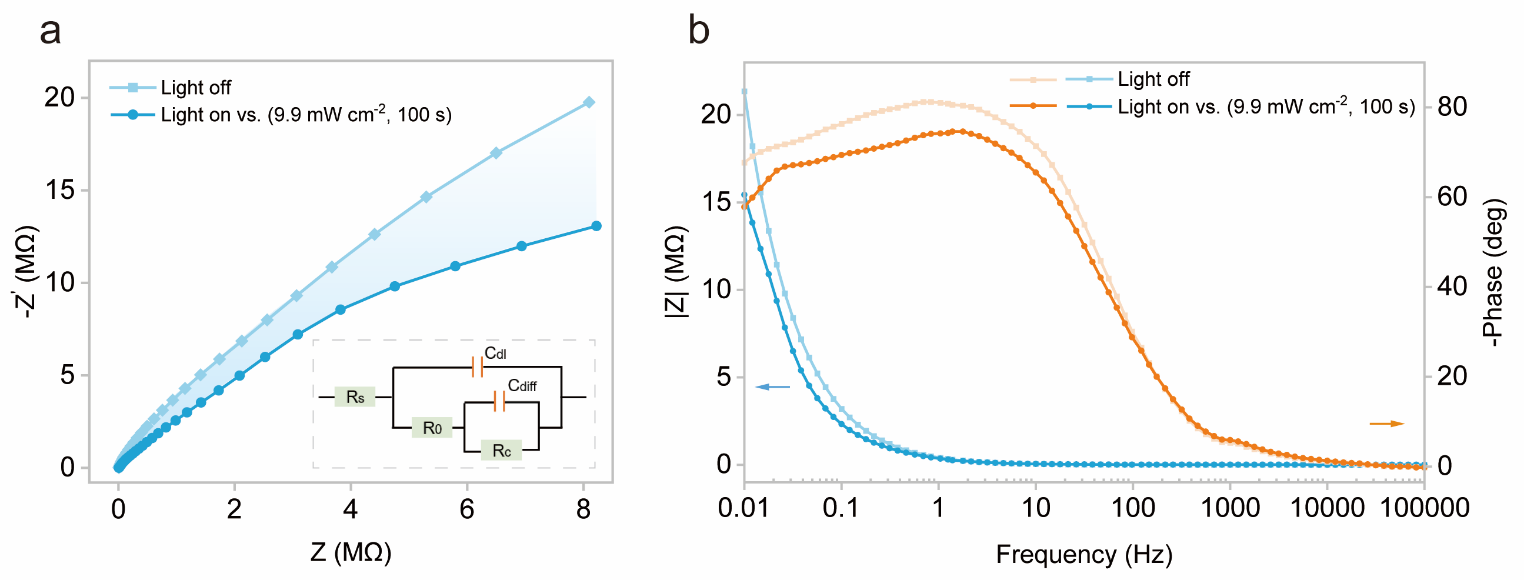
**

**Fig. S6 | a,** Impedance spectrum of the PAPH before and after optical excitation, the illustration is the equivalent circuit in which R_s_ represents equivalent PAPH resistance, C_dl_ and R_0_ represent double layer capacitance and charge transfer resistance at the interface between electrode and hydrogel droplet, C_diff_ and R_c_ represent capacitance and resistance caused by gradient diffusion of charged particles. **b,** Bode diagram of the PAPH device before and after optical excitation.

**
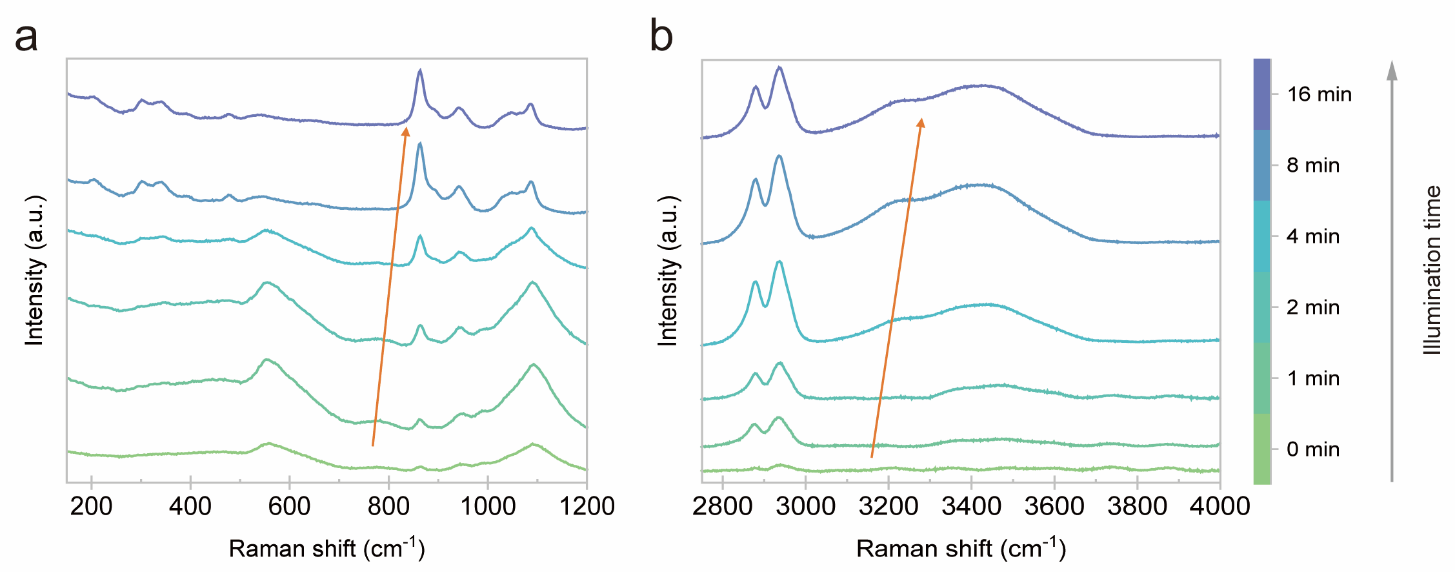
**

**Fig. S7 |** The Raman spectra of negative droplets demonstrated the photochemical process change (Excitation wavelength of 365 nm, Excitation power density of 9.9 mW cm^-2^).

**
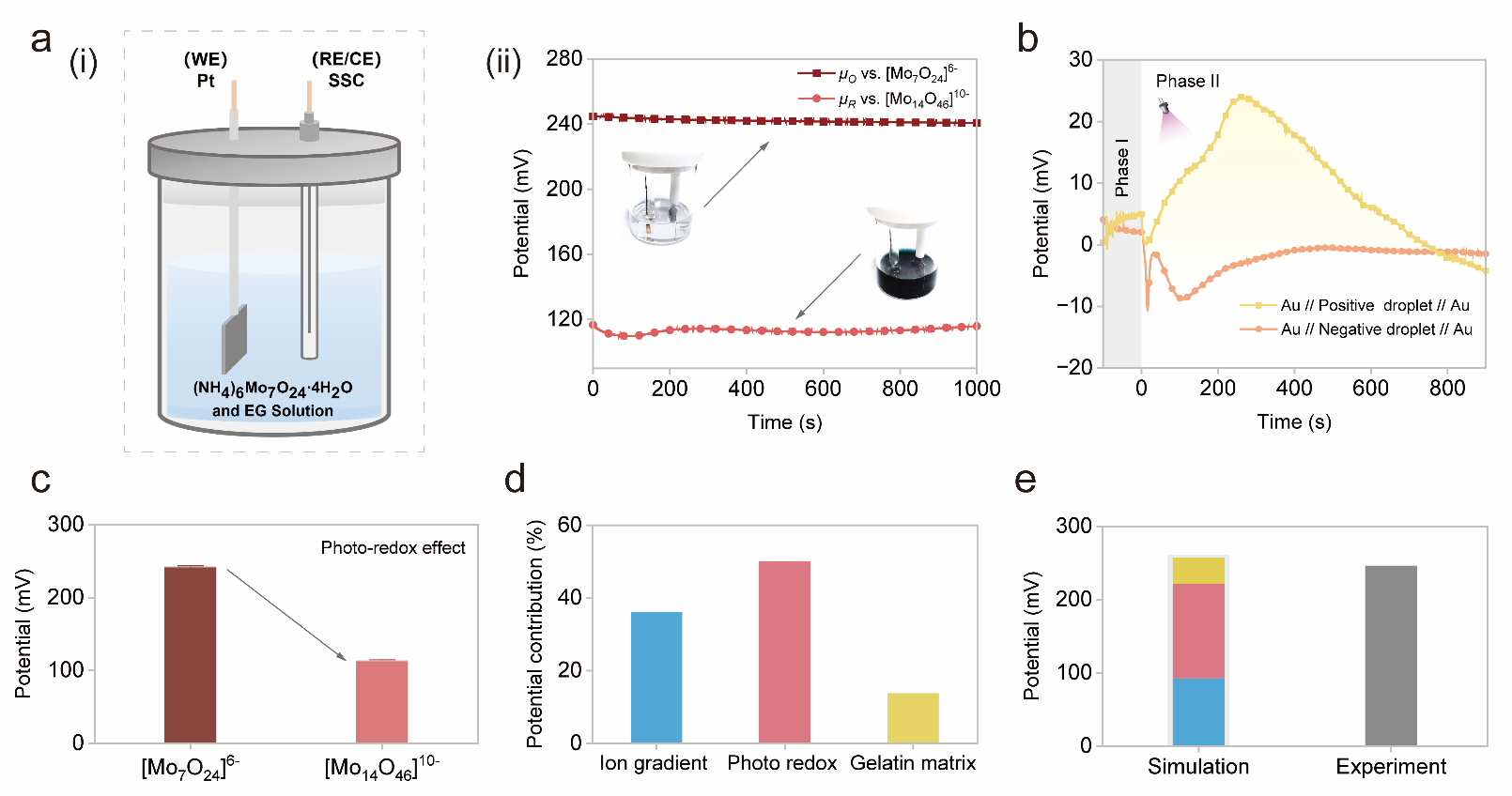
**

**Fig. S8 | The contribution of ion gradient and redox to the open circuit potential in PAPH. a,** The measurement method (i) and the corresponding potential change (ii) for the actual measurement of the redox contribution to the potential under two different states, ${{[Mo}_{14}O_{46}]}^{10-}$ obtained by ${{[Mo}_{7}O_{24}]}^{6-}$after applying the light field (Wavelength of 365 nm, Power density of 9.9 mW cm^-2^) for 300 s, and the test environment is room temperature standard atmospheric pressure. The work electrode (WE) was platinum, whereas SSC (Silver-silver chloride, Ag/AgCl) was used as the reference electrode (RE) and counter electrode (CE), illustration is actual photos of different states used for measurement. **b,** Under the light field, the contribution of gelatin matrix to single positive and negative droplet (Applied at ‘0 s’, Wavelength of 365 nm, Power density of 9.9 mW cm^-2^). **c-e,** The average effect of photo redox on the electrode potential (c), the proportion of various contributions in the photovoltage (d), and the comparison between theoretical and experimental results (e), where the limiting contribution of the ion gradient is calculated by equation (S17), the limiting contribution of the redox potential is determined by testing, and the limiting contribution of the matrix is determined by testing different single droplet.


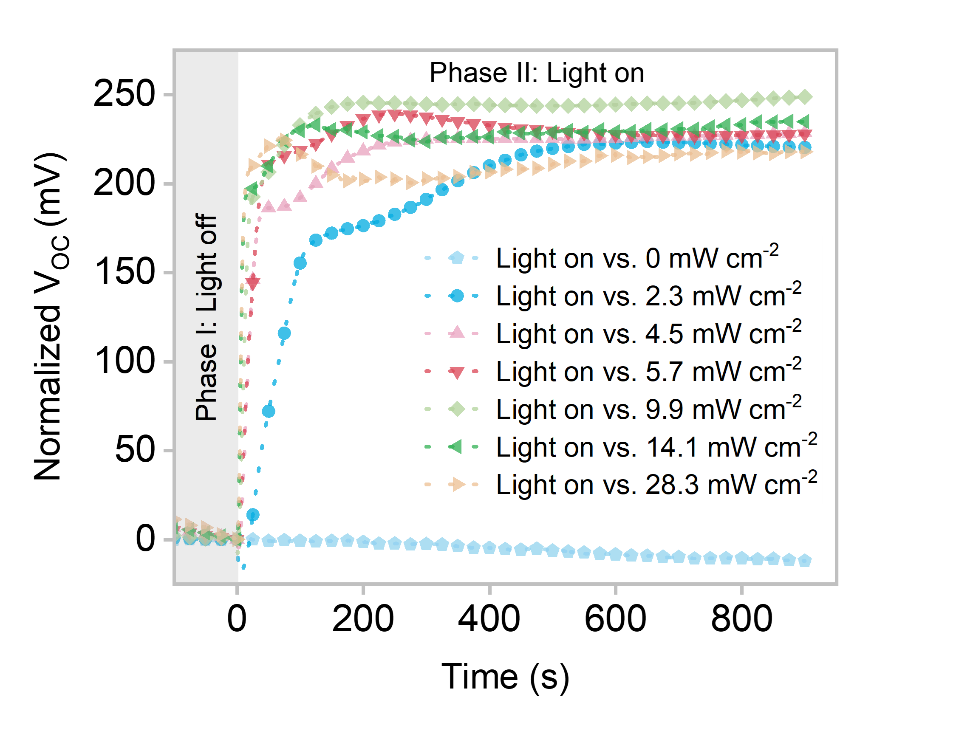


**Fig. S9 |** The normalized output voltage characteristics of PAPH under different optical powers.


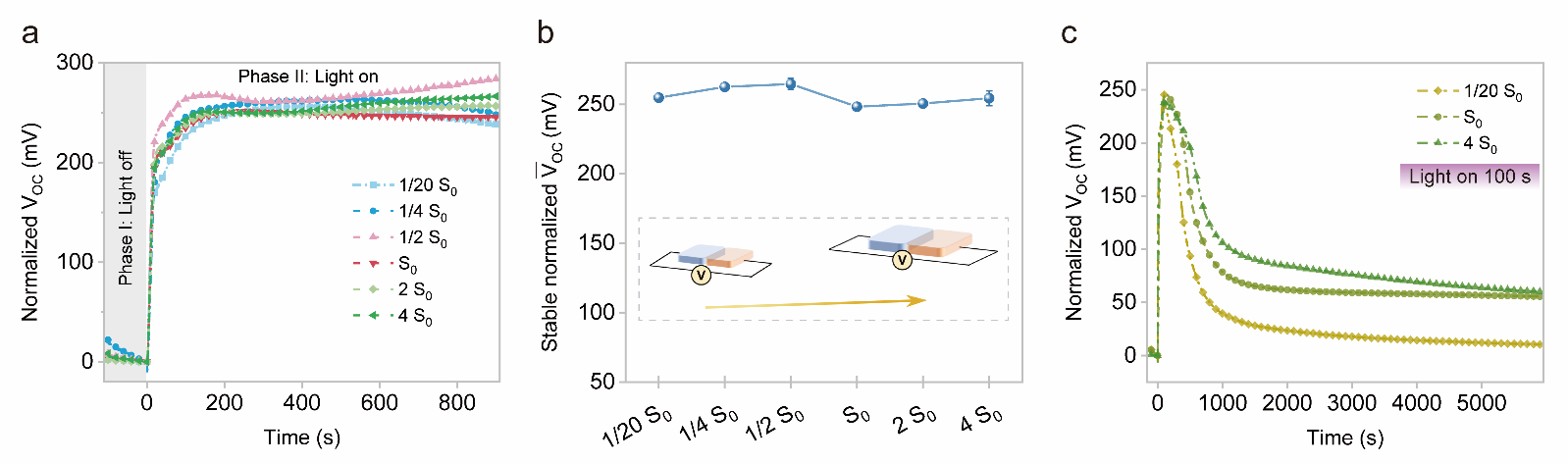


**Fig. S10 | The output characteristics of PAPH with different areas (S_0_ represents the typical unit size of ~10 mm × 10 mm) under the same hydrogel droplet height (~2 mm), and optical power density of 9.9 mW cm^-2^. a,** Normalized open-circuit voltage under continuous illumination. **b,** Steady-state voltage exhibits. **c,** The output characteristics after illumination for 100 s and removal the light.

The output characteristics of PAPH with different areas under the same hydrogel droplet height show in **Fig. S10**. It can be observed that differences caused by volume or shape are also reflected in the steady-state voltage gain. However, since the variations in photochemical processes is not significant, the normalized steady-state voltage exhibits little fluctuation. Upon removal of light excitation, the decay curves show significant differences due to changes in the volume or shape of PAPH, as these alterations may affect the ion diffusion kinetics ^17,18^.

**
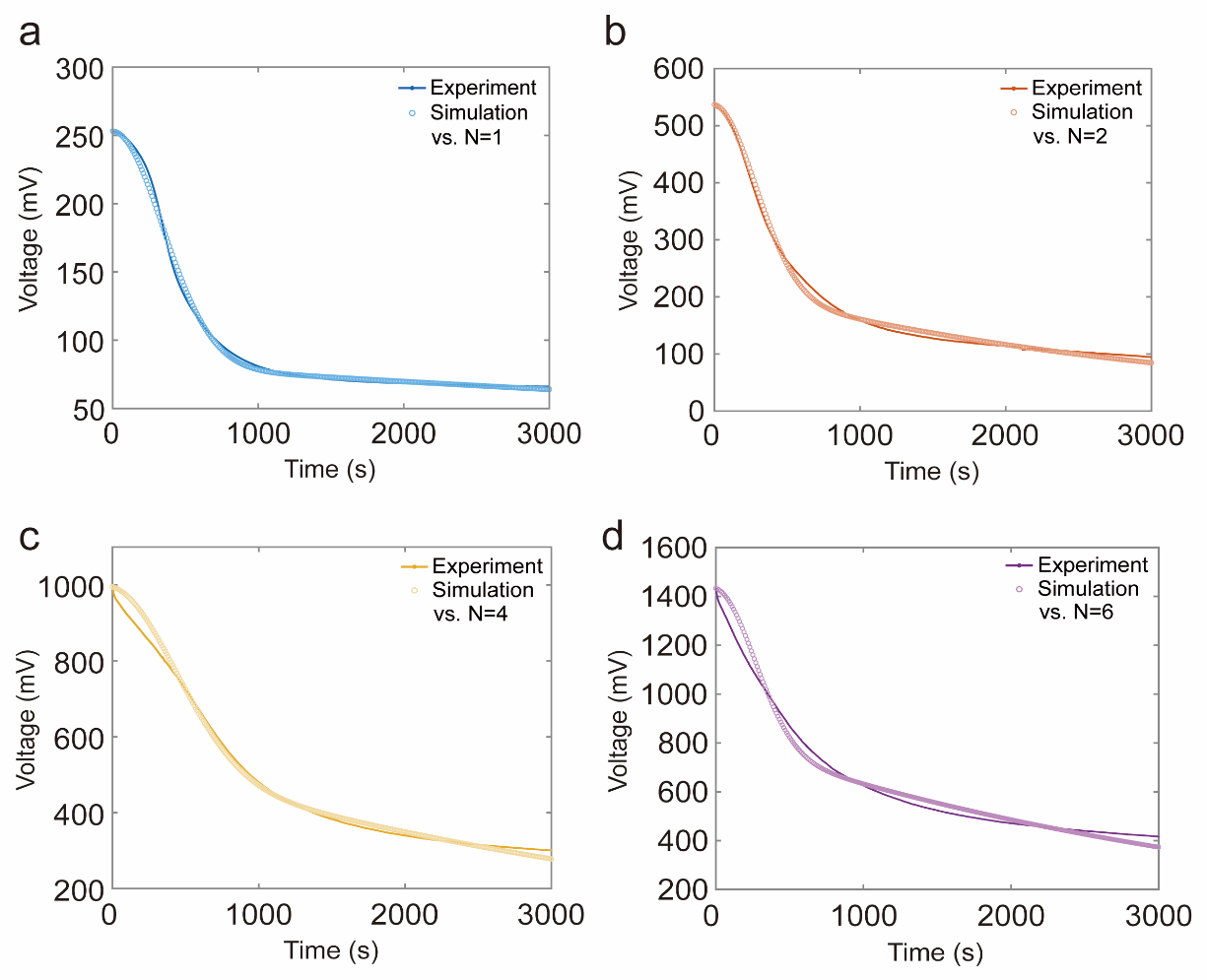
**

**Fig. S11 | The PAPH networks output attenuation fitting after removal of excitation light field (Optical power of 9.9 mW cm^-2^ and illumination for 100 s).** After light off, analysis of experimental results and model fitting results. Number of N=1, 2, 4, 6, as show in a-d, and the parameter fitting results as show in **Table. S1**. Fitting model: $V\left( t \right)=(V_{0}-a_{1}){\times e}^{(-\frac{t}{c_{1}})}{+ a_{1}\times e}^{(-\left( \frac{t}{c_{2}} \right)^{2})}$, where are respectively represented ion diffusion action and photo redox pairs (PR) action. We defined the PR contribution index of $a_{1}$, ion lifetime of $c_{1}$ and half wave width in Gaussian model: $2\sqrt{\ln\left( 2 \right)} \times c_{2}$represents PR change extent.

**Table. S1 |** The parameters fitting results.

| Number | $V_{0}$/mV | $a_{1}$/mV | $c_{1}$/s | $c_{2}$/s |
| --- | --- | --- | --- | --- |
| N=1 | 253.10 | 170.20 | 11550 | 482.60 |
| N=2 | 537.14 | 316.50 | 3134.0 | 402.40 |
| N=4 | 955.48 | 445.10 | 4404.0 | 622.10 |
| N=6 | 1433.2 | 611.20 | 3801.0 | 373.90 |

The PAPH’s ion lifetime for N=2, 4 and 6 is similar, and shorter than for N=1. This is because the PAPH’s ion diffusion trend is towards the positive electrode, cell in series may provide a binding force, leading to a more concentrated ion diffusion direction. The PR change extent is comparable, indicating that of the redox pair recovery degree is similar.

**
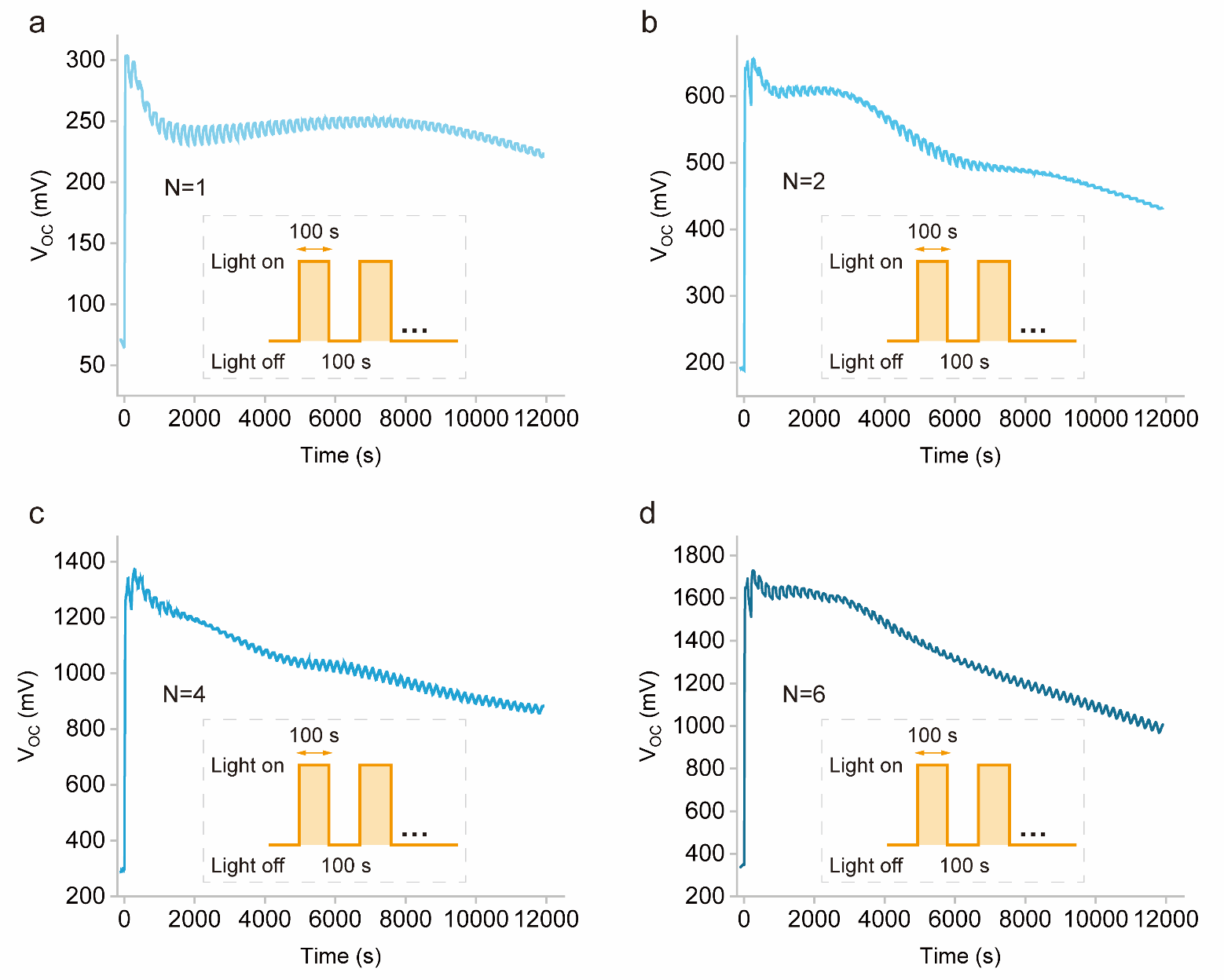
**

**Fig. S12 |** The PAPH networks output characteristics of periodically excited optical field (T=200 s, optical power of 9.9 mW cm^-2^), cell number of N=1 show in (a), N=2 show in (b), N=4 show in (c) and N=6 show in (d).

**
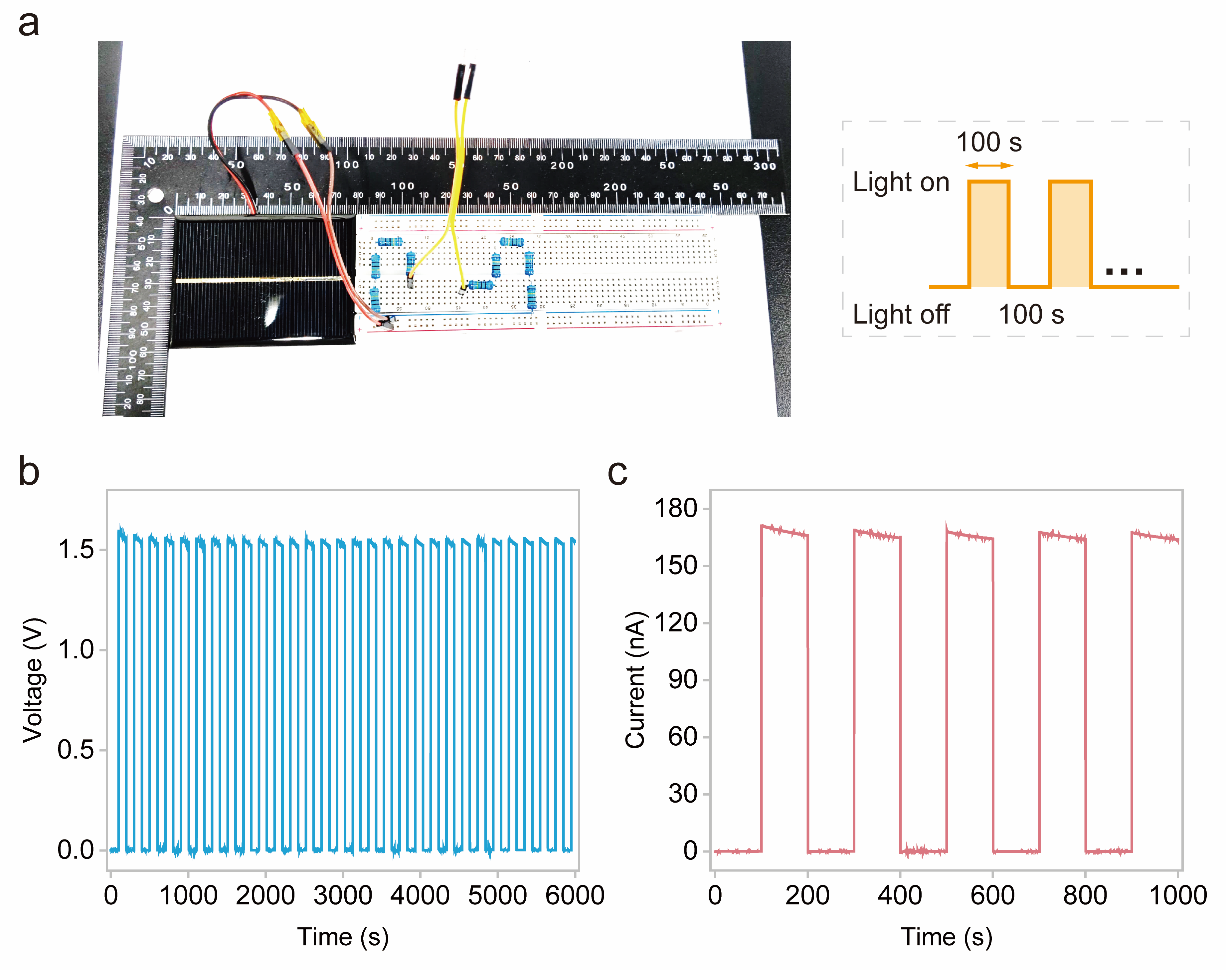
**

**Fig. S13 |** **a,** Photo of a commercial photovoltaic device for cell stimulation experiments, and the light excitation conditions are the same as the PAPH. **b-c,** Voltage output and current output of the device under periodic light field (T=200 s, optical power of 9.9 mW cm^-2^) show in (b) and (c).

**
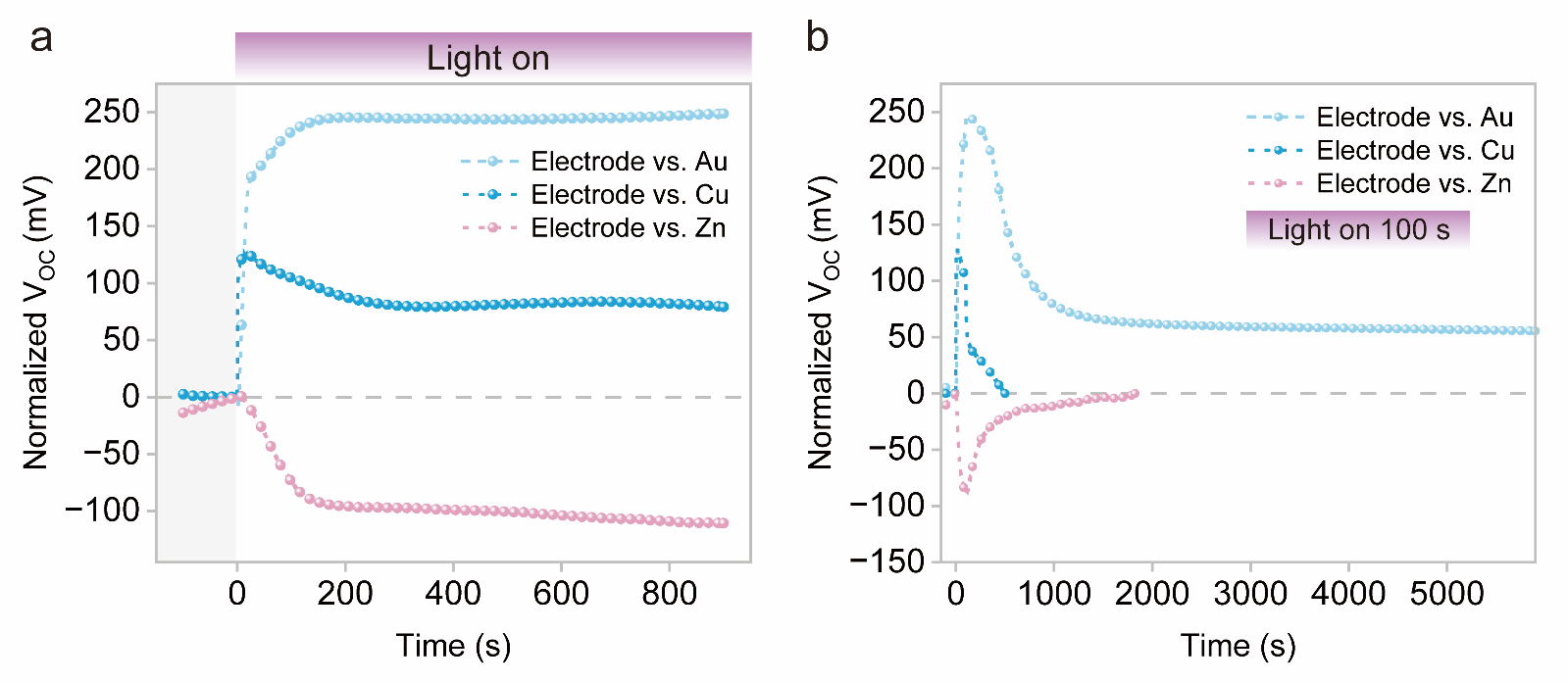
**

**Fig. S14 | Device output** **characteristics of using different electrodes (Optical power of 9.9 mW cm^-2^). a,**$V_{\mathrm{oc}}$ characteristics of different electrodes under continuous light excitation. **b,** Device output attenuation characteristics of using different electrodes after 100 s of light excitation.

The performance of devices with varied electrodes was compared (**Fig. S14**). The electrode activity affects the maximum output and response time of the device, this is because different electrodes exhibit varying responsiveness to ions, which are ultimately manifested in the dual intercalation capacitance difference at the interface between the electrode and hydrogel droplet. However, the primary change trends align with those observed in the typical unit (Construct using Au as the electrode), highlighting the potential versatility and practical application prospects of our designed hydrogel droplet-based light-harvesting device.


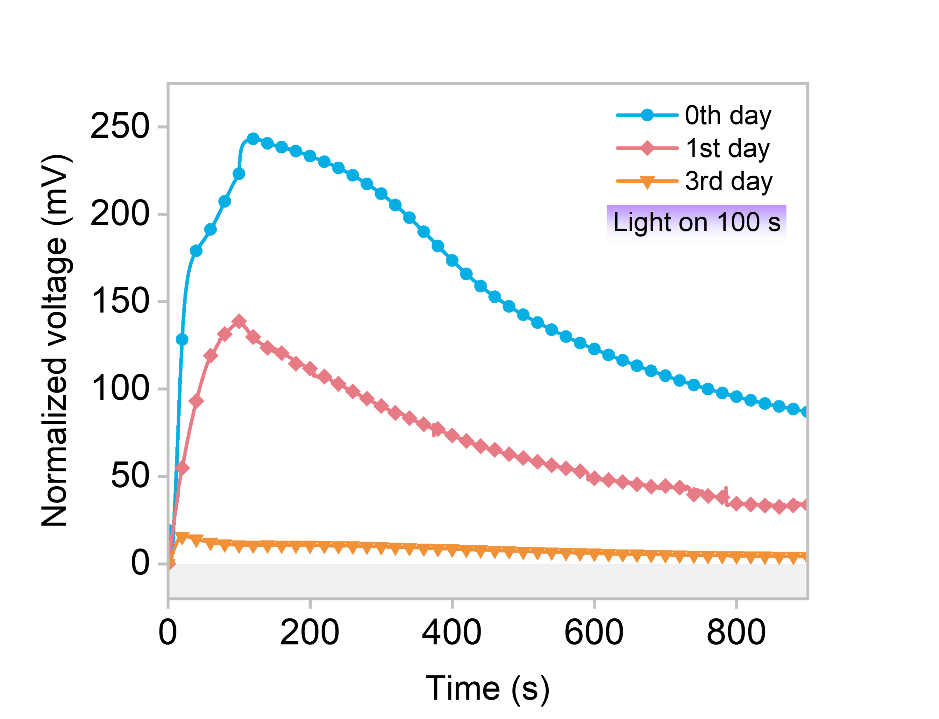


**Fig. S15 |** The normalized voltage characteristics (Optical power density of 9.9 mW cm^-2^) of the same PAPH (Typical unit size of ~10 mm × 10 mm × 2 mm) on the 0th, 1st, 3rd within a different long-term storage (Atmospheric environmental conditions).

We characterized the light harvesting output characteristics of the same PAPH unit at different long-term storage (Atmospheric environmental conditions) on 0th, 1st, and 3rd day. The corresponding normalized voltage curves are shown in **Fig. S15**. Under initial conditions, it can be observed that a typical PAPH unit can provide a voltage gain of ~250 mV after 100 s of photoenergy harvesting. After one day, the curve characteristics altered, and the peak voltage gain exhibited approximately 45 % degradation, which can likely be attributed to irreversible ion diffusion and minor moisture loss. Following three days, the voltage gain performance underwent significant degradation, likely caused by substantial moisture loss, thereby impeding the effective execution of photochemical processes and interactions with the electrodes. For the PAPH hydrogel droplet device we designed, by introducing materials exhibiting effects opposite to those of molybdate ions in photochemical processes (Just like dual-ion batteries) ^19,20^, combined with lipid coating encapsulation of hydrogel droplets, may serve as an effective strategy to potentially enhance repeatability use or long-term storage performance in future studies.

**Reference**

1 Yamase, T. Photo- and Electrochromism of Polyoxometalates and Related Materials. *Chem Rev* **98**, 307–326 (1998).

2 Wang, Y. F. *et al.* Programmable photo-responsive self-healing hydrogels for optical information coding and encryption. *Eur Polym J* **166**, 111025 (2022).

3 Guan, L. *et al.* Highly transparent and stretchable hydrogels with rapidly responsive photochromic performance for UV-irradiated optical display devices. *React Funct Polym* **138**, 88-95 (2019).

4 Wang, J. *et al.* Ultrasoft gelatin aerogels for oil contaminant removal. *J Mater Chem A* **4**, 9381-9389 (2016).

5 Jiang, J. X. *et al.* A multifunctional gelatin-based aerogel with superior pollutants adsorption, oil/water separation and photocatalytic properties. *Chem Eng J* **358**, 1539-1551 (2019).

6 Rizwan, K. *et al.* Recent advancements in engineered biopolymeric-nanohybrids: A greener approach for adsorptive-remediation of noxious metals from aqueous matrices. *Environ Res* **215**, 114398 (2022).

7 He, Z. R. & Yuan, W. Z. Adhesive, Stretchable, and Transparent Organohydrogels for Antifreezing, Antidrying, and Sensitive Ionic Skins. *Acs Appl Mater Inter* **13**, 1474-1485 (2021).

8 Han, C. G. *et al.* Giant thermopower of ionic gelatin near room temperature. *Science* **368**, 1091-1098 (2020).

9 Debye, P. & Hückel, E. The theory of electrolytes. I. Lowering of freezing point and related phenomena. *Physikalische Zeitschrift* **24**, 185-206 (1923).

10 Xu, J. *et al.* Synthetic Protocells to Mimic and Test Cell Function. *Adv Mater* **22**, 120-127 (2010).

11 Schroeder, T. B. H. *et al.* An electric-eel-inspired soft power source from stacked hydrogels. *Nature* **552**, 214-218 (2017).

12 Kapat, K. *et al.* Piezoelectric Nano-Biomaterials for Biomedicine and Tissue Regeneration. *Adv Funct Mater* **30**, 1909045 (2020).

13 Zhang, X. Y. *et al.* Biomedical Applications of Electrets: Recent Advance and Future Perspectives. *J Funct Biomater* **14**, 320 (2023).

14 Li, L. *et al.* Design and Applications of Photoresponsive Hydrogels. *Adv Mater* **31**, 1807333 (2019).

15 Song, E. *et al.* Materials for flexible bioelectronic systems as chronic neural interfaces. *Nat Mater* **19**, 590-603 (2020).

16 Xiao, A. *et al.* A Degradable Bioelectronic Scaffold for Localized Cell Transfection toward Enhancing Wound Healing in a 3D Space. *Adv Mater* **36**, 2404534 (2024).

17 Zhang, Y. J. *et al.* A microscale soft ionic power source modulates neuronal network activity. *Nature* **620**, 1001-1006 (2023).

18 Jastram, A. *et al.* Swelling and Diffusion in Polymerized Ionic Liquids-Based Hydrogels. *Polymers-Basel* **13**, 1834 (2021).

19 Xu, W. H. *et al.* Some basics and details for better dual-ion batteries. *Energ Environ Sci* **18**, 2686-2719 (2025).

20 Zhang, L. J. *et al.* A Review of Emerging Dual-Ion Batteries: Fundamentals and Recent Advances. *Adv Funct Mater* **31**, 2010958 (2021).
